# The Independent Probabilistic Firing of Transcription Factors: A Paradigm for Clonal Variability in the Zebrafish Retina

**DOI:** 10.1016/j.devcel.2015.08.011

**Published:** 2015-09-14

**Authors:** Henrik Boije, Steffen Rulands, Stefanie Dudczig, Benjamin D. Simons, William A. Harris

**Affiliations:** 1 Department of Physiology, Development and Neuroscience, Cambridge University, Cambridge CB2 3DY, UK; 2 Department of Physics, Cambridge University, Cambridge CB3 0HE, UK; 3 Department of Neuroscience, Uppsala University, 751 24 Uppsala, Sweden

## Abstract

Early retinal progenitor cells (RPCs) in vertebrates produce lineages that vary greatly both in terms of cell number and fate composition, yet how this variability is achieved remains unknown. One possibility is that these RPCs are individually distinct and that each gives rise to a unique lineage. Another is that stochastic mechanisms play upon the determinative machinery of equipotent early RPCs to drive clonal variability. Here we show that a simple model, based on the independent firing of key fate-influencing transcription factors, can quantitatively account for the intrinsic clonal variance in the zebrafish retina and predict the distributions of neuronal cell types in clones where one or more of these fates are made unavailable.

## Highlights

• A simple quantitative model can explain clonal variability in the retina
• This model is based on the firing probabilities of key transcription factors
• These probabilities are shown to be largely independent of each other
• The environment has only a minor effect on these probabilities

## Graphical Abstract

**Figure:**
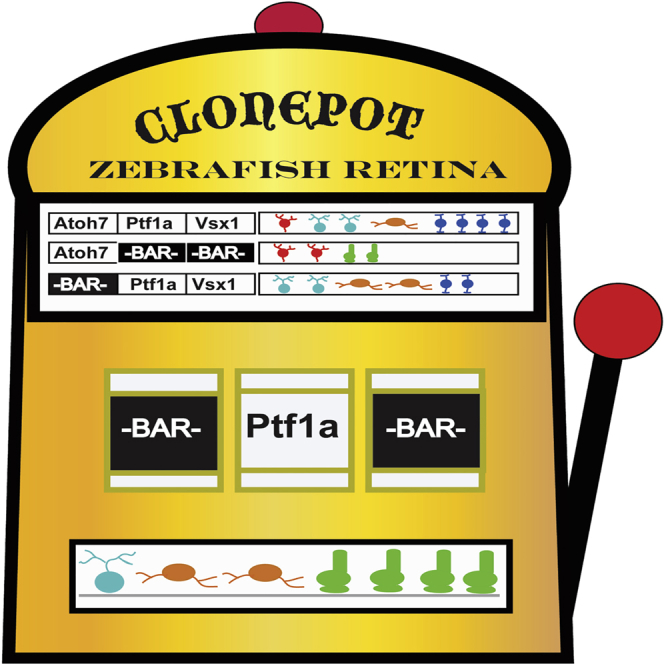

## Teaser

Boije et al. show that a simple model, based on independent and probabilistic firing of key fate-influencing transcription factors, can quantitatively account for intrinsic clonal variance in zebrafish retina. Robust distributions generated through stochastic processes can explain how one retina is almost invariant from the next despite high clonal variability.

## Introduction

It is estimated that the human brain contains over 100 billion cells of more than 10,000 different types (Azevedo et al., 2009). Understanding how all of these cells are generated in the correct proportions is one of the great challenges of developmental neuroscience. To address this question, it is critical to investigate how individual CNS progenitors generate clones of mature neurons. In the vertebrate CNS, it is known that retinal progenitor cells (RPCs) at the optic cup stage are multipotent and give rise to clones that are highly variable both in size and neuronal fate composition (Holt et al., 1988; Turner and Cepko, 1987; Wetts and Fraser, 1988). The finding that clones derived from isolated individual rat RPCs grown in vitro are just as variable as those in vivo, suggests that this variability is an intrinsic property of RPCs (Cayouette et al., 2003). One possibility is that these RPCs are individually programmed to go through unique and determined lineage trees. An alternative possibility, however, is that early RPCs are essentially equivalent but that probabilistic mechanisms drive differences in clonal sizes and compositions. The latter hypothesis is supported by recent studies on rat and zebrafish RPCs, where it has been shown that simple stochastic models can accurately account for the clone size distributions and lineage patterns (Gomes et al., 2011; He et al., 2012).

**Figure 1:**
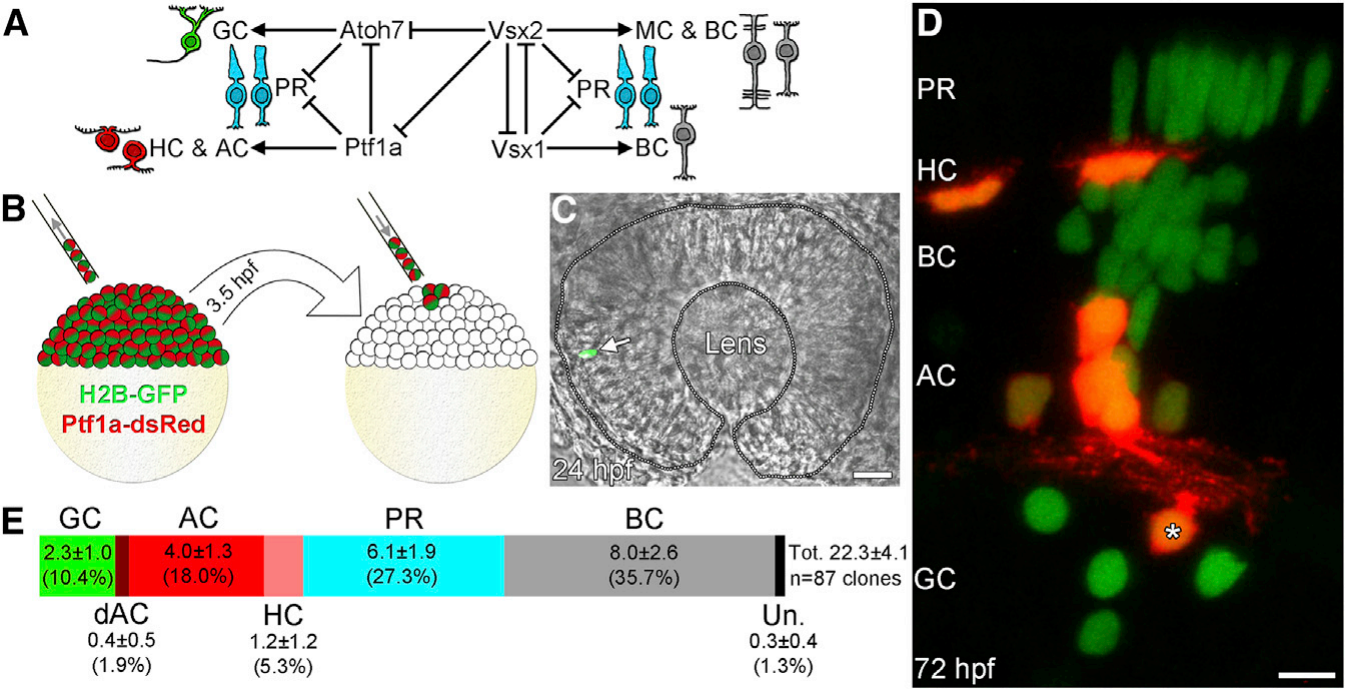
Blastomere Transplantation Allows Clonal Analysis of RPCs (A) A core network of four key TFs can explain much of the cellular diversity in the retina. (B) Cells from H2B-GFP, Ptf1a-dsRed double transgenic embryos were transplanted into WT embryos at 3.5 hpf. (C) Embryos were screened for isolated RPCs at 24 hpf. (D) At 72 hpf differentiation is completed with radial clones generated by transplanted cells. The asterisk marks a dAC. (E) Quantification of cell fate distribution in clones generated by WT RPCs into WT hosts. Cell numbers and SDs are indicated as are the percentages of an average clone. Un, unknown. Scale bars represent 20 μm in (C) and 5 μm in (D). See also Tables S1 and S2.

Work from many laboratories has uncovered a gene regulatory network (GRN) of key transcription factors (TFs) that control some of the earliest cell fate decisions among the five main neuronal cell types of the vertebrate retina (Figure 1A; reviewed in Boije et al., 2014; Xiang, 2013). This GRN is activated when the repressive TF, Vsx2, is downregulated in RPCs, thus releasing these cells to express various fate-specifying TFs (Burmeister et al., 1996; Levine and Green, 2004; Vitorino et al., 2009). The first of these is the bHLH TF, Atoh7, which is necessary and sufficient for the generation of ganglion cells (GCs) (Hernandez et al., 2007; Kanekar et al., 1997; Liu et al., 2001; Prasov et al., 2012; Vetter and Brown, 2001; Yang et al., 2003). Vsx2 downregulation also de-represses FoxN4, which turns on Ptf1a, a TF that is necessary and sufficient for the generation of amacrine cells (ACs) and horizontal cells (HCs), and is capable of overriding Atoh7’s GC-promoting activity (Dullin et al., 2007; Fujitani et al., 2006; Jusuf et al., 2011; Lelièvre et al., 2011; Vitorino et al., 2009). Some Ptf1a expressing cells co-express Lhx1, and these adopt HC fates (Boije et al., 2013; Lelièvre et al., 2011). Other cells, released from Vsx2 repression, express Vsx1 and give rise to the majority of bipolar cells (BCs) in the zebrafish retina (Chow et al., 2001; Ohtoshi et al., 2001; Vitorino et al., 2009). Finally, a small proportion of RPCs in the zebrafish retina re-express Vsx2 and give rise to Muller cells (MCs) and a single subclass of BCs, distinct from the subclasses that express Vsx1 (Burmeister et al., 1996; Livne-Bar et al., 2006; Vitorino et al., 2009). Within this GRN, photoreceptors (PRs) can be considered as a default fate (Dorval et al., 2006; Le et al., 2006; Toy et al., 2002). While the exploration of this GRN has revealed several of the earliest TFs involved in cell fate diversification in the retina, little light has been shed on how Atoh7, Ptf1a, Lhx1, and Vsx1 come to be expressed in a way that ensures that all of the main retinal cell types are generated in consistent proportions.

It seemed possible that the probabilistic firing of the genes encoding the TFs released from Vsx2 repression in this network could explain the variability of fate distributions within clones. To test this idea, we developed a simple model based on the assumption that these TFs fire probabilistically and independently of each other within a set of equipotent RPCs. We then tested this model against a large array of clonal datasets from RPCs in which we deliberately perturbed the probabilities of expressing each of these factors individually. In all cases, this model was capable of making good predictions about the distributions of cellular compositions and sizes of clones arising from these RPCs. We thus conclude that the independent and probabilistic expression of these TFs is capable of explaining most of the variance in cell type composition seen in zebrafish retinal clones.

## Results

### Generation of Clones

In order to generate retinal clones in which all cell types could be identified based on nuclear position and reporter gene expression, blastomeres were transplanted from H2B-GFP; Ptf1a-dsRed double-transgenic zebrafish embryos into WT embryos at 3.5 hr post fertilization (hpf) (Figure 1B). H2B-GFP labels all nuclei while Ptf1a-dsRed is expressed in cells destined to become HCs or ACs (Jusuf and Harris 2009). The cell-cycle of RPCs during optic cup formation is very slow but speeds up by at least a factor of four at about 24 hpf (Li et al., 2000), making this an ideal time point to screen host embryos for single, isolated, GFP-labeled cells in the optic cup (Figure 1C). We also found pairs of labeled cells that appeared to be derived from a single progenitor that had recently divided (see Supplemental Experimental Procedures). However, as we did not actually witness the divisions generating these pairs, we classified single-cell origin and two-cell origin clones separately. By 72 hpf, central retinal development is complete with radial clones generated by the transplanted cells, allowing quantification of their size and fate composition (Figures 1D and 1E; Tables S1 and S2). The cell fate distribution in these clones agrees well with previously published fate distributions in the zebrafish retina (He et al., 2012).

### Modeling Cell Fate Distributions

To derive a model for the generation of clonal cell type variability, we began by suggesting that the downregulation of Vsx2 allows RPCs to express the key TFs in this fate specifying GRN with certain fixed probabilities. The three TFs we consider here are Atoh7, Ptf1a, and Vsx (which includes Vsx1 and Vsx2) (Figure 1A) (Chow et al., 2001; Fujitani et al., 2006; He et al., 2012; Jusuf et al., 2011; Le et al., 2006; Vitorino et al., 2009; Yang et al., 2003). The model is simply based on the idea that the genes encoding these key TFs in this GRN fire probabilistically and independently of each other.

RPCs progress through three distinct phases starting with a proliferative phase in which cells divide symmetrically, termed PP type. The proliferative phase is followed by the first neurogenic phase in which RPCs choose between all three modes of division, PP, PD and DD, according to defined probabilities, and a late neurogenic phase in which the predominant mode of division is terminal, DD (He et al., 2012; Livesey and Cepko, 2001). Atoh7 is upregulated prior to mitosis in PD divisions leading to one differentiating and one proliferative cell (He et al., 2012), while Ptf1a is expressed immediately following mitosis (He et al., 2012; Jusuf and Harris, 2009; Poggi et al., 2005). Based on this behavior and additional studies that suggest that the TFs Atoh7, Ptf1a, and Vsx are expressed during restricted time windows, we sought to define the simplest model of fate choice that is compatible with this observed progression (Boije et al., 2008; Brzezinski et al., 2012; Decembrini et al., 2009; Vitorino et al., 2009).

**Figure 2:**
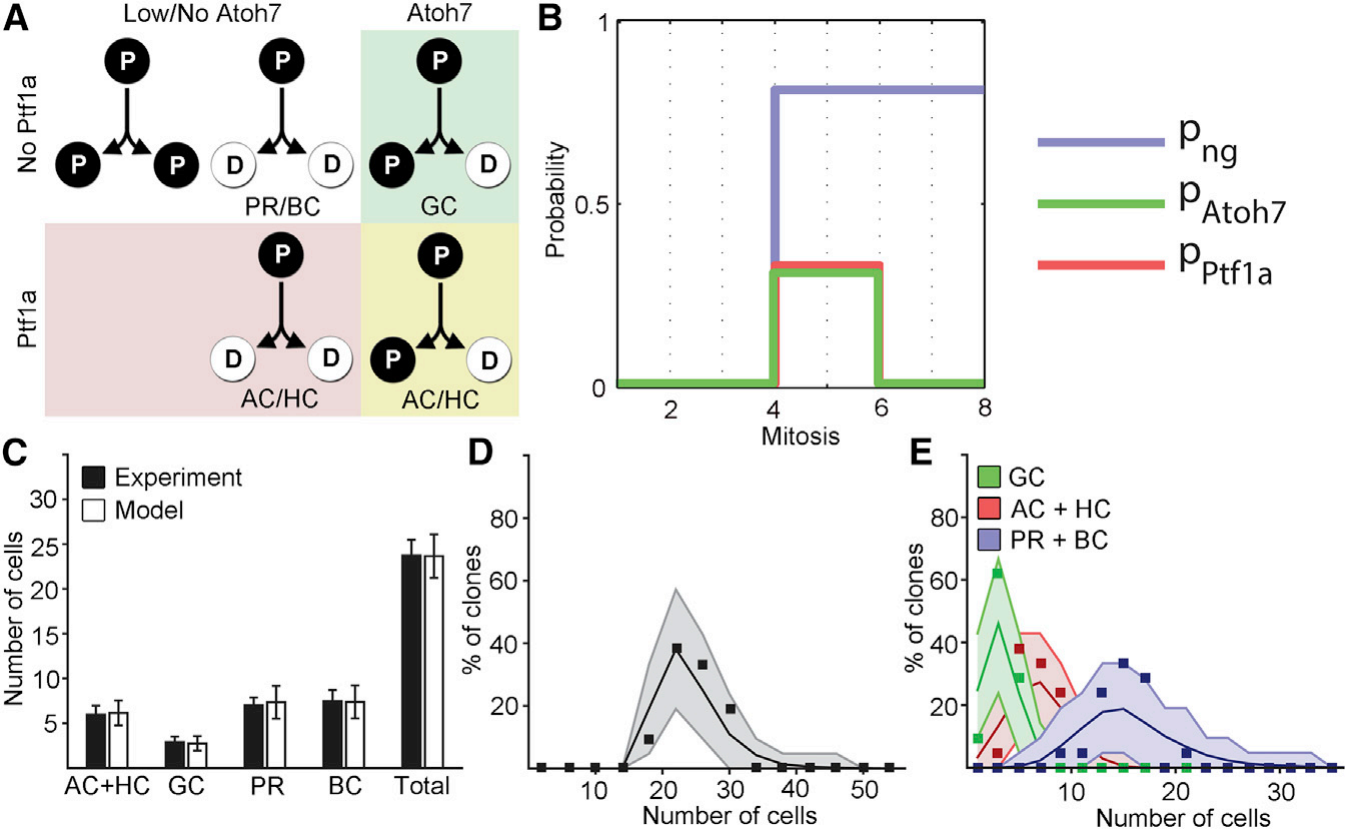
Clone Size and Cell Fate Distributions of Retinal Clones Can Be Recapitulated by a Minimal Model (A) Combinatorial expression of Ptf1a and Atoh7 gives rise to four distinct groups that adopt fates differently, and where cells either continue to proliferate (P) or differentiate (D). (B) Temporal progression of the probabilities of expressing Atoh7, Ptf1a and undergoing a neurogenic division (p_ng_). (C) Averages of the fate distributions generated in the experimental clones compared with a set of virtual RPCs allowed to flow through the model. Error bars depict SDs. (D) Clone size distribution of binned experimental data (black boxes) compared with the model. Note that in this and in similar figures the shaded regions denote the expected variation (95% confidence intervals) around the theoretical curves due to the limited experimental sample size. (E) Cell fate distributions in experimental clones compared with the model. For visualization reasons PRs and BCs are merged but individual distributions are available in Figure S2. P, proliferative progenitor; D, differentiated cell. See also Table S4.

The model (shown in Figure 2) initiates at the time when RPCs in the retina begin to cycle rapidly at around 24 hpf. After three rounds of symmetric PP divisions, the eight descendants of a RPC reach the first neurogenic phase where they may upregulate the two TFs, Ptf1a and Atoh7, with fixed probabilities (i.e., *p*_*Atoh7*_, *p*_*Ptf1a*_) (Figure 2B). This creates four classes of RPCs (Figure 2A): those that express Atoh7 but not Ptf1a and as a result generate GCs through PD divisions; those that express of Ptf1a but not Atoh7 and generate ACs and HCs through terminal DD divisions; those that express both Atoh7 and Ptf1a leading to the production of ACs and HCs through PD divisions; and finally, those that express neither Atoh7 nor Ptf1a. Cells in this last class produce BCs and PRs through terminal DD divisions or remain proliferative (PP). As there are an almost equal numbers of PRs and BCs in the zebrafish retina, we suggest that the decision between these two fates can be effectively described as a “coin flip.” Half of these differentiating cells will express Vsx and become BCs, while the other half will become PRs. We define one more parameter, which reflects the probability of differentiating (the neurogenic probability, *p*_*ng*_). After three cell cycles in the first phase of neurogenesis, any remaining RPCs enter a second neurogenic phase, where *p*_*Atoh7*_ and *p*_*Ptf1a*_ both drop to zero while *p*_*ng*_ remains unchanged (see Supplemental Experimental Procedures).

Our next challenge was to assign fixed values to *p*_*Atoh7*_, *p*_*Ptf1a*_, and *p*_*ng*_. Previous studies of clone size distributions in zebrafish found that, in late-stage retinal development, roughly 80% of divisions are of DD type, which translates to *p*_*ng*_ = 0.8 (He et al., 2012). With *p*_*ng*_ constrained, we were then left with just two parameters to fit from analysis of the experimental data. We calculated these by minimizing the sum of the squared errors between the mean cell numbers predicted by the model and the corresponding mean values obtained in the above clonal dataset, from WT RPCs in WT hosts. Thus, *p*_*Atoh7*_ = 0.32 ± (0.04, 0.03), and *p*_*Ptf1a*_ = 0.30 ± (0.04, 0.05) (Figure 2B). With all parameters fixed by calibration to the WT mean values, we then asked whether a theoretical set of RPCs following the model dynamics could produce a set of clones that match not only the means but also higher moments of the experimental distributions. Significantly, as well as capturing the average abundances of the various cell types (Figure 2C), this simple model also provides an excellent fit to the detailed distributions for both cell numbers and cell fates (Figures 2D, 2E, and S2; Table S4).

### Testing the Independent Firing of TFs

A basic prediction of our model is that when the choice of a particular cell type becomes unavailable due to the knockdown of a single TF, cells must choose among all the other available cell types in accordance with set probabilities. Specifically, the model posits that *p*_*Atoh7*_ and *p*_*Ptf1a*_ are specified independently of each other, and if this assumption is right, the independent probabilistic model should be able to predict the distributions of clone sizes and compositions from RPCs in which these factors are individually knocked down. To test the model experimentally, we transplanted blastomeres from H2B-GFP; Ptf1a-dsRed embryos injected with morpholinos targeting Atoh7, Ptf1a, or Vsx1 into WT hosts, thus keeping the environment constant.

**Figure 3:**
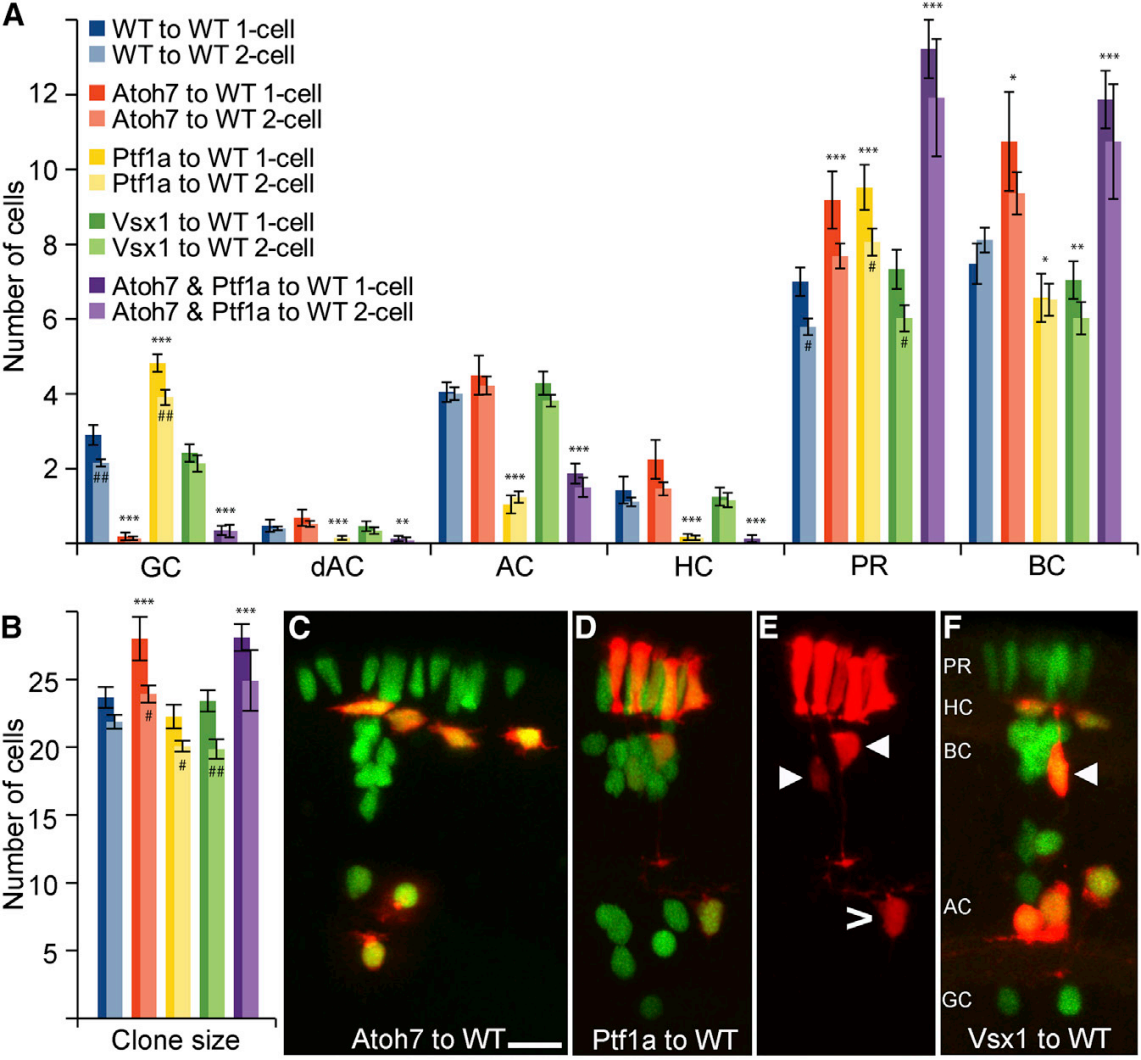
Intrinsic Impact on Cell Fate (A) Fate distribution of clones generated by Ptf1a, Atoh7, or Vsx1 morphant cells transplanted into WT hosts. ^∗^ indicates significance for the merged p values of one- and two-cell compared with WT, while # denotes significant difference between one- and two-cell clones within a particular morphant. ^∗^/# p < 0.05, ^∗∗^/## p < 0.01, ^∗∗∗^/### p < 0.001. Error bars depict SEM. The figure legend in (A) is also valid for (B). For the number of clones for the different treatments, see Table S1, and for statistical calculations, see Table S3. (B) Average clone sizes generated from single RPCs scored at 24 hpf for the various morphants in WT environment. (C–F) Representative micrographs of clones generated by Atoh7, Ptf1a, or Vsx1 morphant cells in a WT environment, respectively. The red channel is shown individually for the Ptf1a morphant clone in (E) to reveal the fate switch performed by the Ptf1a lineage to PRs, BCs (marked by arrow head) and GCs (marked by hollow arrow head). Arrowhead in (F) indicates a Ptf1a-positive BC. The scale bar in (C) represents 10 μm and is also valid for (D)–(F). See also Figure S4 and Table S2.

**Figure 4:**
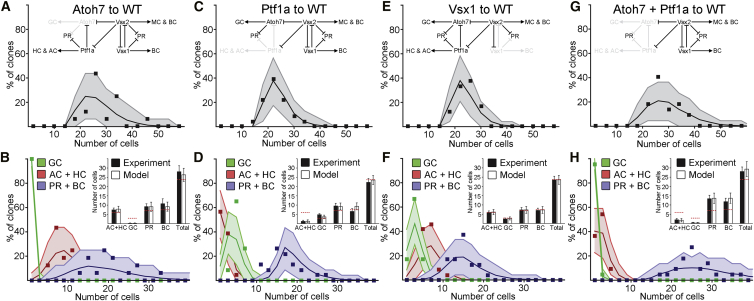
Modeling of Intrinsic Factors (A and B) Modeling of clone size (A) and fate distribution (B) of Atoh7 morphant clones in WT hosts. Inset in (A) depicts the part of the network that has been primarily affected in the donor RPCs. Inset in (B) depicts averages of the experimental values and the values from the modeling with corresponding SDs. Dotted red line represents the WT value. (C and D) Modeling of clone size and fate distribution of Ptf1a morphant clones in WT hosts. (E and F) Modeling of clone size and fate distribution of Vsx1 morphant clones in WT hosts. (G and H) Modeling of clone size and fate distribution of Atoh7, Ptf1a double morphant clones in WT hosts. See also Figure S7 and Table S4.

Clones derived from RPCs of Atoh7 morphant embryos resulted in a major (93%) reduction in the number of GCs (Figures 3A and 3C; Table S3). Concomitantly, there was an increase in the average number of ACs/HCs, PRs, and BCs as well as a significantly larger average clone size (Figures 3A, 3B, and S3A). To test the model against the experimental data, we simply reduced the probability *p*_*Atoh7*_ by the same 93%, while the other parameters remained unchanged. We accounted for the fact that RPCs that would have undergone asymmetrical PD divisions, due to Atoh7 expression, now undergo symmetric PP divisions (He et al., 2012). The model then faithfully recapitulates the data (Figures 4A and 4B; Table S4). Tellingly, while the number of ACs/HCs was increased, the proportion of ACs/HCs within these clones was not significantly different (Figures S3A and S3B). This suggests that the additional cells, generated by the increase in PP-divisions, have the same probability of expressing Ptf1a as WT RPCs. Also, the 50/50 split between PRs and BCs observed in WT clones was preserved following the increase in both populations due to the loss of GCs, strongly suggesting that the reduction in *p*_*Atoh7*_ did not affect the probability of expressing Vsx (Figure 4B, inset).

Ptf1a morphants displayed a 79% decrease in ACs and HCs, allowing us to estimate *p*_*Ptf1a*_ = 0.06 for these RPCs (Figures 3A, 3D, and 3E). The reduced probability of making ACs and HCs in morphant clones, according to our model, should translate into an increased probability of making other cell types, and indeed, these clones showed such increases (Figure 3A). As expected, Ptf1a morphant clones showed no significant change in clone size compared with WT clones, suggesting that Ptf1a knockdown does not significantly affect the distribution of division modes as is reflected in the model (Figure 3B). Assuming that Ptf1a knockdown does not produce additional PP divisions, the model again does a good job at predicting the distributions of cell fates and cell numbers (Figures 4C and 4D; Table S4).

We also looked at clones generated from Vsx1 morphant RPCs in a WT environment. However, as Vsx1 and Vsx2 are reciprocally repressive, the reduction of Vsx1-positive BCs leads to a compensating increase in Vsx2-positive BCs (Figures S4A and 4B). Thus, the model, which treats Vsx1 and Vsx2 as equivalent TFs, and the data agree well with each other and with the results from WT RPCs (Figures 3A, 3B, 3F, 4E, and 4F). While Vsx2-positive BCs are all of the same S4 subtype in WT embryos with a single terminal button stratifying in the IPL (Connaughton and Nelson, 2000; Vitorino et al., 2009), the Vsx2-positive BCs in Vsx1 morphant clones stratify in multiple layers with varying complexity, as is seen within the Vsx1 lineage (Figures S4A and S4B). Thus, the probability of expressing either Vsx1 or Vsx2 appears to make no difference to the probability of expressing Atoh7 or Ptf1a. Moreover, the ratio of BCs and PRs, which is linked to the expression of Vsx, remains constant at one-half in all these datasets, which strongly suggests that the expression of Vsx is independent of Ptf1a and Atoh7.

To challenge the model even further, we asked whether it could predict size and fate distributions of clones generated by Atoh7, Ptf1a double morphant RPCs, i.e., RPCs in which GC, AC, and HC fates are compromised. Our data show that such clones are significantly larger than WT clones and contain, as expected, primarily PRs and BCs (Figures 3A and 3B). We modeled the double knockdown by reducing the probabilities of expressing Atoh7 and Ptf1a by the same amounts as estimated for single morphants individually. The model again does a good job of predicting clone size and fate redistributions (Figures 4G and 4H; Table S4). Thus, in all the cases that we examined, the experimental data strongly support the suggestion that the cells of clones in which a particular fate is unavailable distribute themselves among the remaining fates in a manner that is consistent with the stochastic rules of the model. Considering the fact that *p*_*Atoh7*_ and *p*_*Ptf1a*_ were calibrated only against the WT means and that all these distributions were deduced without further fitting, we find it striking that the experimental distributions match the theoretical predictions so well.

To further challenge the basis of the independent probabilistic model, we then compared its behavior to two alternative models based on a component of interdependent TF expression (see Supplemental Information). In the first alternative model, Ptf1a and Atoh7 are positively interdependent; i.e., the knockdown of Ptf1a leads to a knockdown in Atoh7 and vice versa. In the second alternative model, Ptf1a and Atoh7 are negatively interdependent; i.e., the knockdown of Ptf1a leads to overexpression of Atoh7 and vice versa. As the probabilities of TF factor expression in all these models were derived from the means of the WT to WT dataset, it is not surprising that all three models fit the WT to WT dataset (Figure S7). However, both interdependent models fail when they are asked to predict clonal distributions derived from Atoh7 and the Ptf1a morphant RPCs (Figure S7). This analysis shows that a simple model of independent TF expression does a good job at explaining the experimental outcomes in the different treatments, whereas introducing an interdependent component makes the predictions worse (Figure S7). Taken together, these results strongly support the view that the independent probabilistic expression of TFs is the simplest modeling paradigm capable of predicting these experimental distributions.

### The Generation of HCs Is Consistent with the Independent Probabilistic Expression of TFs

**Figure 5:**
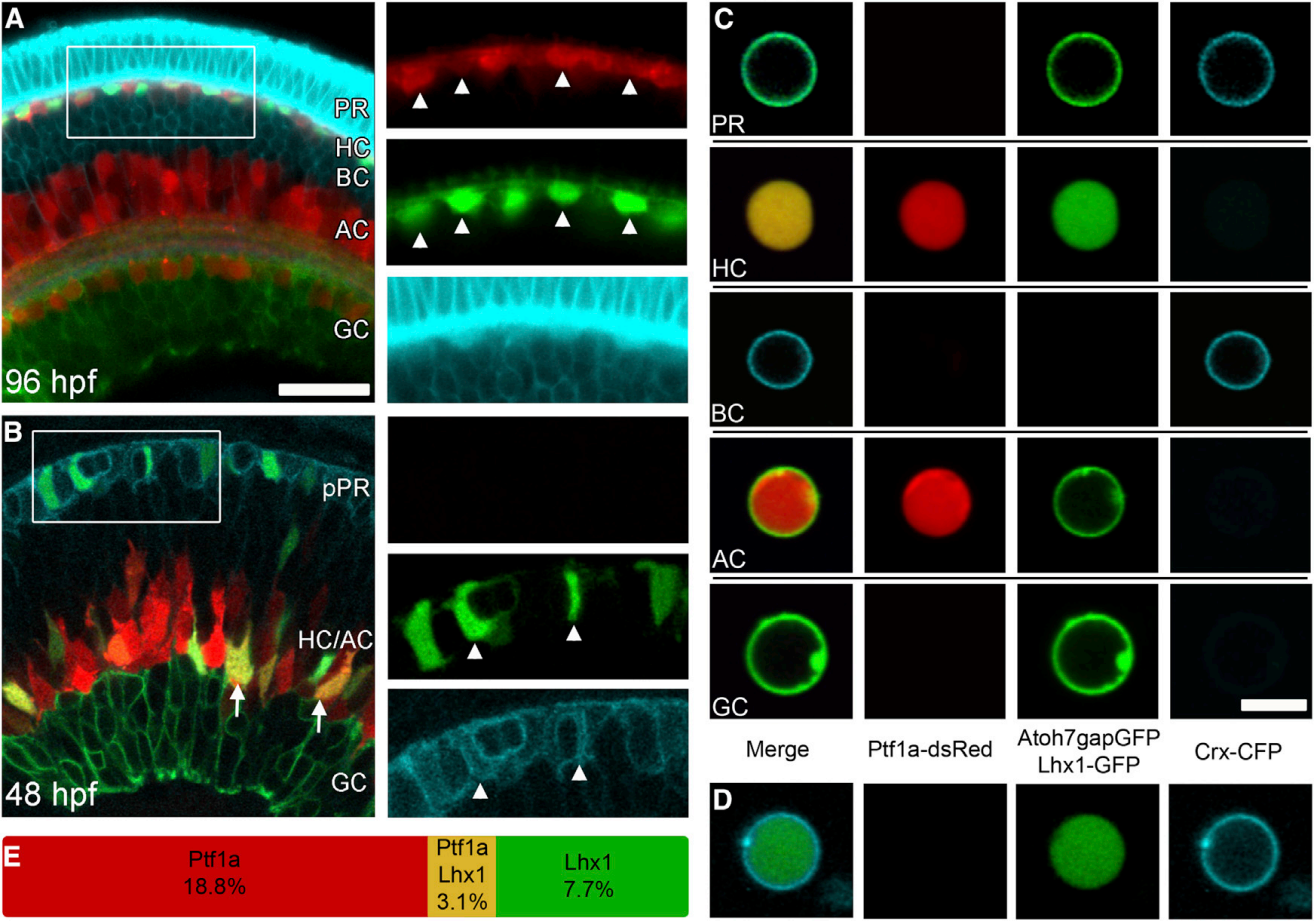
HCs Are Generated as the Intersection between the Lhx1 and Ptf1a Lineages (A) The retina of a quadruple transgenic zebrafish (Atoh7-gapGFP; Ptf1a-dsRed; Lhx1-GFP; Crx-gapCFP) at 96 hpf. Individual channels with arrowheads illustrating the overlap between GFP and dsRed. (B) Quadruple transgenic retina at 48 hpf. Arrows indicate Lhx1-GFP, Ptf1a-dsRed double positive cells, i.e., HCs. Individual channels reveal overlap between Crx-CFP and Lhx1-GFP, as indicated by arrowheads. pPR, putative PR. (C) Combinatorial expression in the quadruple transgenic allows identification of the different cell types in a dissociated sample. Channel designation below is valid for both (C) and (D). (D) Lhx1-positive PR cell. (E) Percentages of cells that express Lhx1, Ptf1a, or both Lhx1 and Ptf1a. The scale bar in (A) represents 20 μm and is also valid for (B), while the scale bar in (C) represents 5 μm and is also valid for (D).

As currently defined, our simple model does not deal with ACs and HCs as separate populations. To assess whether independent probabilistic expression of TFs could be a feature of the HC fate decision, we began by investigating the quantitative relationship between Lhx1 and Ptf1a expression. It has previously been noted that Lhx1 is expressed in a subpopulation of HCs (Edqvist et al., 2006; Lelièvre et al., 2011; Suga et al., 2009). We found, however, that all HCs are generated from the Lhx1 lineage within the larger Ptf1a-positive population (Figure 5A). We also found that there are numerous Lhx1-positive cells, which are not Ptf1a positive, that become PRs (Figures 5B and 5D). If the probabilities of expressing Ptf1a and Lhx1 are independent of each other, then the proportion of cells that are HCs (i.e., the population of cells that express both TFs) should simply translate to the product of these two probabilities.

To find these probabilities, we used quadruple transgenic embryos (Atoh7-gapGFP; Ptf1a-dsRed; Lhx1-GFP; Crx-gapCFP), which allowed us to accurately quantitate the expression of Ptf1a and Lhx1 in dissociated cells at 72 hpf (Figures 5C and 5D). Our counts from four separate experiments revealed that 20.9% ± 2.3% of WT retinal cells expressed Ptf1a and 10.8% ± 1.1% expressed Lhx1, with a predicted intersectional population, assuming independent expression, of 2.3% ± 0.47%. We found that 3.1% ± 0.55% of the dissociated cells expressed both Ptf1a and Lhx1 (i.e., are HCs), which is not statistically different from the predicted percentage (Figure 5E). This finding is therefore consistent with the model paradigm for cell fate specification conditioned by the independent probabilistic expression of corresponding key TFs.

### A Minor Influence of Extrinsic Feedback

**Figure 6:**
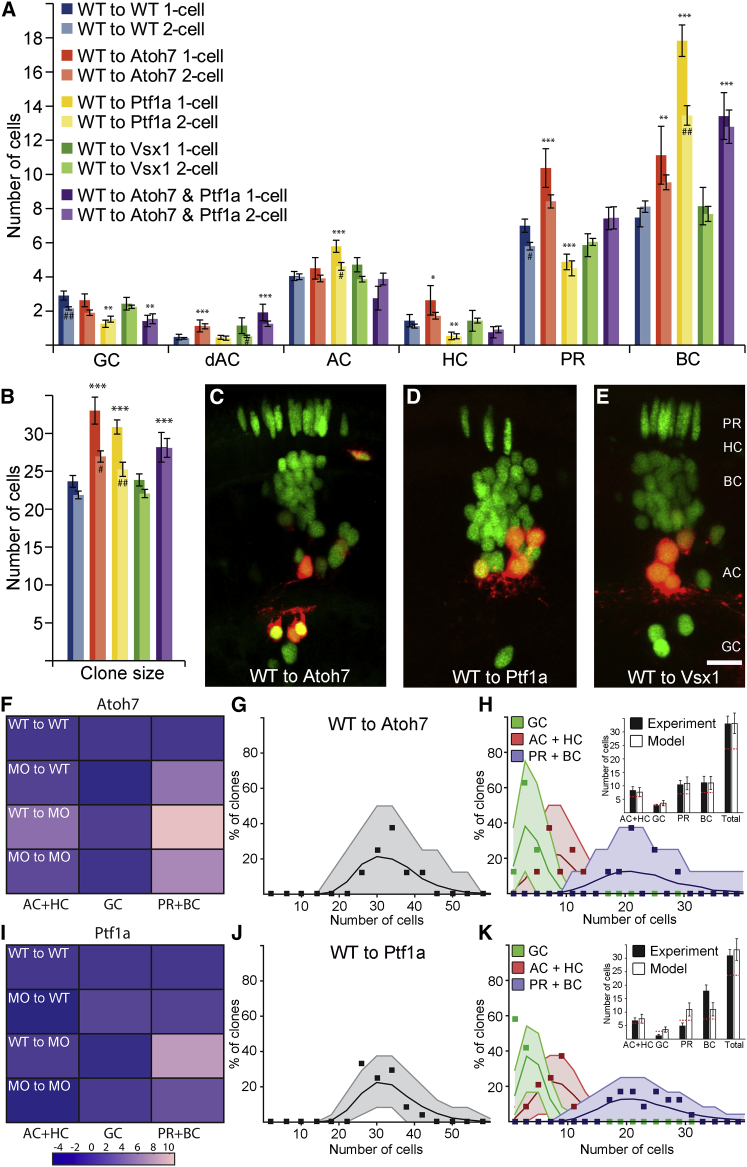
Extent of Extrinsic Feedback during Retina Development (A) Fate distribution of clones generated by WT cells transplanted into Ptf1a, Atoh7, Vsx1 or Atoh7, Ptf1a double morphant hosts. ^∗^ indicates significance compared with WT, while # denotes significant difference between one- and two-cell clones within a particular morphant. Figure legend is also valid for (B). Error bars depict SEM. (B) Average clone size generated from single RPCs scored at 24 hpf for WT cells and the various environments previously described. (C–E) Representative micrographs of WT clones generated in Atoh7, Ptf1a, or Vsx1 morphant environments, respectively. (F) Checker-plot visualizing the extent of intrinsic regulation and extrinsic feedback in the different Atoh7 morphant scenarios. Color denotes deviation from WT to WT, such that a brighter color corresponds to an increase while a darker corresponds to a decrease of cell numbers of a given cell type. (G and H) The intrinsic model is modified by the introduction of a delay postponing the onset of neurogenesis in 60% of the RPCs by one division. The resulting clone size (G) and fate (H) distributions match the experimental clones. Inset depicts averages of the experimental values and the values from the modeling with corresponding SDs. (I–K) As in (F)–(H) but for the Ptf1a morphant environment with the same delay introduced to the intrinsic model. The scale bar in (E) represents 10 μm and is also valid for (C) and (D). See also Figures S5 and S6 and Table S5.

Several studies suggest that the retinal environment fine tunes fate assignments (Yang, 2004). To look for extrinsic influences, we simply reversed the experimental situation and transplanted WT RPCs, marked by H2B-GFP and Ptf1a-dsRed expression, into different morphant or mutant environments (Figures 6 and S6). Previous experiments in which WT cells were transplanted into *lak* mutant zebrafish, which have a mutation in the Atoh7 gene, indicated an increase in GCs in such clones (Poggi et al., 2005). Unexpectedly, in the present study, using Atoh7 morphants as hosts, we did not observe this homeostatic compensation of GCs, but found instead simply a larger average clone size (Figures 6A–6C). To see whether the discrepancy was due to the use of morphant rather than mutant hosts, we also analyzed clones generated by WT RPCs transplanted into *lak* mutant retinas, but the results were essentially identical (Figures S6A and S6B). Interestingly, we also observed HCs displaced in the GC layer in morphant hosts, suggesting that the abnormal plexiform layer formation in these embryos may be responsible for trapping both ACs and HCs (Figures S5E–S5G; Table S5).

Similar experiments with WT RPCs in Ptf1a morphant retinas revealed similar extrinsic effects. Compared with WT clones in a WT environment, WT clones in Ptf1a morphant retinas were larger (Figures 6A, 6B, and 6D). However, in this case, there was a clear underproduction of GCs. As extrinsic signaling from GCs is thought to inhibit further production of GCs, the increased numbers of GCs in the Ptf1a MO hosts seemed like a possible explanation for this underproduction of GCs in the transplanted WT RPCs. However, transplantation of WT RPCs into Atoh7, Ptf1a double morphant hosts in which there are no GCs in the host, revealed the same reduction in GCs generated from the transplanted WT RPCs ruling out this hypothesis (Figures 6A and 6B). As the Ptf1a morpholino only prevents roughly 70% of the ACs from being formed, we also transplanted WT cells into retinas that were further deprived of ACs and HCs, generated by injection of a mixture of two different Ptf1a translation blocking morpholinos. This Ptf1a morpholino mixture eliminates ∼95% of all ACs and HCs, which should further reduce the amount of feedback from generated HCs and ACs (Randlett et al., 2013). WT clones in such retinas are, however, similar to those in which the single morpholino was used (Figures S6A, S6B, and S6G).

Interestingly, we also noted an apparent reduction in HCs, but no general difference in the number of Ptf1a-positive cells in WT clones that developed in Ptf1a morphant hosts (Figures 6A and S3A). To see whether some of Ptf1a-positive cells in the AC layer were HCs, we transplanted cells from Lhx1-GFP; Ptf1a-dsRed expressing donors into Ptf1a morphant hosts and found that many HCs in these clones reside in the AC layer (Figures S5A–S5C; Table S5). Since all HCs initially migrate to the AC layer before migrating apically toward the OPL (Edqvist and Hallböök, 2004), the fact that intrinsically WT HCs do not make this migration in the morphant hosts suggests the existence of an extrinsic signal. We suggest that ACs might be the origin of this external signal as the failure of HC migration is inversely proportional to the number of ACs in the transplanted clones, the only source of ACs in these otherwise AC-less retinas (Figures S5D and S5G).

We also transplanted WT H2B-GFP; Ptf1a-dsRed cells into Vsx1 morphant hosts, but here we found no significant extrinsic effects on fate composition or clone size when comparing to WT clones in WT hosts (Figures 6A, 6B, and 6E). This is not surprising considering that the reciprocal repression of Vsx1 and Vsx2 means that the loss of Vsx1 is largely compensated by the upregulation of Vsx2 resulting in little change in the number BCs or indeed of any of the main cell types in the morphant retinas.

The similar increase in clone sizes and change in fate distribution observed both in the Atoh7 and Ptf1a morphant environments suggests that there might be a unified explanation for these extrinsic influences (Figures 6F and 6I). This prompted us to see whether a single minor adjustment of the model could account for this effect. Indeed, we were able to describe most of the changes that occur in both morphant environments by postponing the onset of neurogenesis by approximately half a cell cycle, i.e., by assuming that 60% of the RPCs enter neurogenesis one division later than they do in a WT environment (Figures 6G, 6H, 6J, and 6K). The ability of the model to largely predict the complex clonal distribution and compositional data following such a major change in the environmental input provides confidence that the basic model captures the key regulatory machinery.

### Lineages in Whole Knockdown Retinas: Combining Intrinsic Potential with Extrinsic Influence

In many knockout studies, both the progenitor cells and the environment they develop in are mutant, making it difficult to quantitatively account for intrinsic versus extrinsic effects. To understand what happens in a retina when the expression of particular fate influencing gene is lost or knocked down in a whole animal, we have to consider mutant or morphant RPCs developing within a mutant or morphant environment, i.e., both the intrinsic and extrinsic influences on retinal lineages. To test whether the model that incorporates the extrinsic effect (viz. the delay in neurogenesis) can predict fate distributions in such scenarios, we transplanted morphant cells from H2B-GFP; Ptf1a-dsRed transgenic embryos into unlabeled morphant hosts. By comparing deviations (Figures 6F and 6I), it is clear that morphant clones in morphant hosts are roughly similar to morphant clones in WT hosts, showing that intrinsic influences dominate in these experiments.

**Figure 7:**
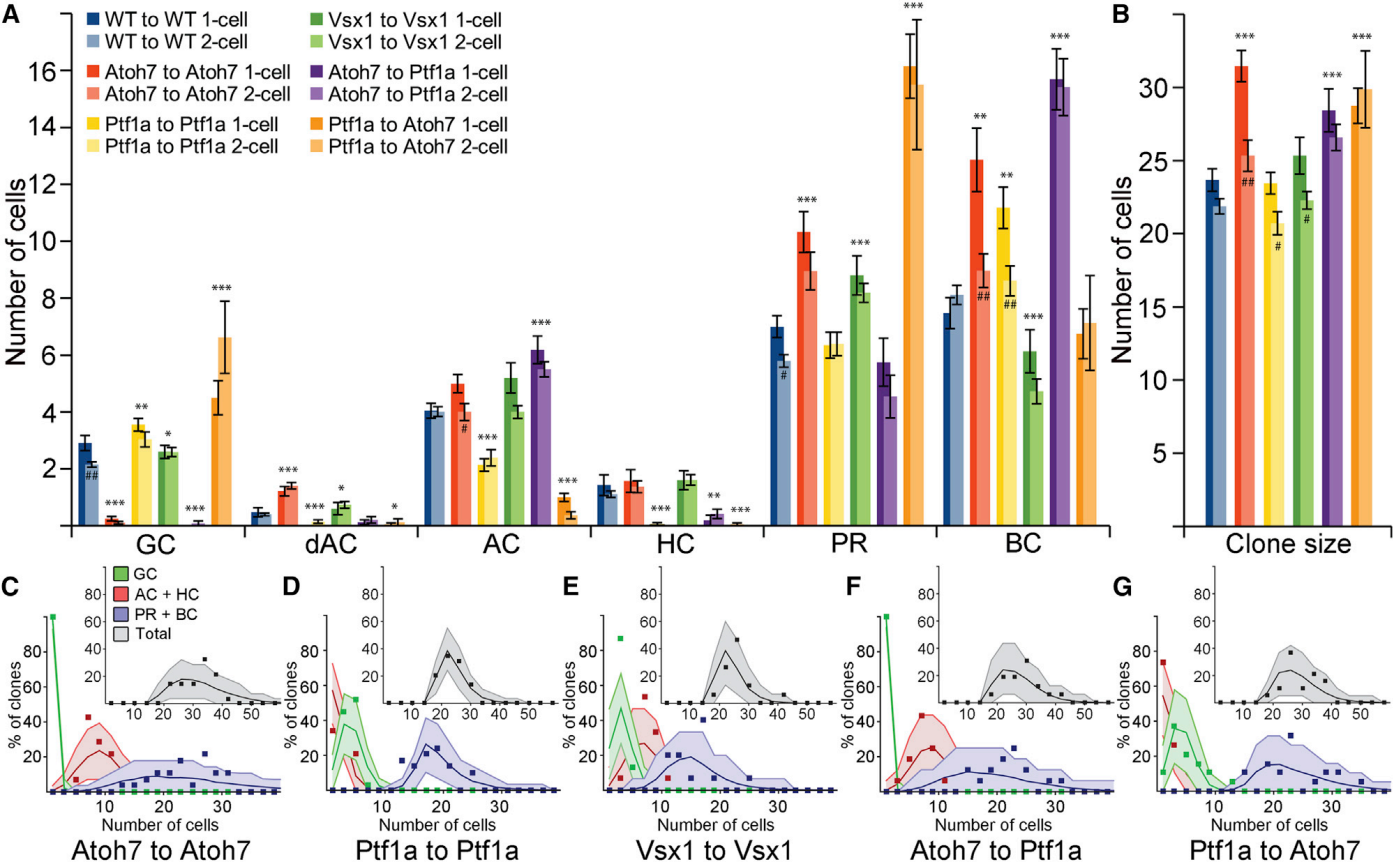
Combining the Intrinsic and Extrinsic Effects (A) Fate distribution of clones generated by morphant cells transplanted into morphant hosts. ^∗^ indicates significance compared with WT, while # denotes significant difference between 1- and 2-cell clones within a particular morphant. Figure legend is also valid for (B). Error bars depict SEM. (B) Average clone size generated from single RPCs scored at 24 hpf for morphant cells in morphant environments. (C–G) Predicted distributions of clone sizes and cell types obtained by combining the intrinsic and extrinsic theory for various morphant conditions. For individual distributions, see Figure S2. See also Figure S6 and Tables S4 and S6.

Experimentally, Atoh7 morphant clones in an Atoh7 morphant environment show a large decrease in the frequency of GCs and an increased frequency of all other cell types, accompanied by an increase in total clone size (Figures 7A and 7B). These results are consistent with clonal data generated by an alternative assay in which WT and Atoh7 morphant embryos had single cells in Maze-Kaede transgenic retinas photoconverted (see Supplemental Experimental Procedures), allowing us to quantify clonal expansion from single RPCs (Figures S6C–S6F; Table S6).

We found that, while the purely intrinsic model generated distributions similar to the experimental clones, we could favorably increase the quality of the fit in the Atoh7 morphant by adding the delay in neurogenesis due to the extrinsic effect of the morphant environment (Figure 7C; Table S4). In the case of Ptf1a morphant clones within a Ptf1a morphant host, the full model also fits the data well, but not significantly better than the intrinsic model (Figure 7D). For Vsx1 morphant clones in Vsx1 morphant retinas, as there are no discernable extrinsic effects, the purely intrinsic model continues to fit the data well (Figures 7A, 7B, and 7E). Interestingly, we also challenged the model to predict clonal distributions in cross-morphant scenarios, i.e., clones derived from Atoh7 morphant RPCs in Ptf1a morphant environments or vice versa. Again, the description for the cross morphant clones followed straightforwardly by combining the extrinsic effects found for the WT to morphant transplantation with the intrinsic model; in the case of Atoh7 morphant clones in a Ptf1a morphant environment, both the intrinsic and combined model worked equally well, while in the case of Ptf1a morphant clones in an Atoh7 morphant environment, the model incorporating the delay due to the extrinsic effect does a better job at predicting clonal distributions than the purely intrinsic model (Table S4).

## Discussion

The findings above show that the variability in the clonal composition seen in the zebrafish retina can be quantitatively explained by the probabilistic and independent firing of fate influencing TFs. The independent nature of TF expression here means that when one of these TFs is reduced in RPCs, new clonal distributions can be predicted by the unchanged probabilities of the other TFs firing, which we show is also the case. Indeed, the changes in the fraction of total cells expressing Atoh7 and Ptf1a in WT and morphant Spectrum of Fate lines of zebrafish (Almeida et al., 2014 and unpublished data) are in good agreement with the results presented here. Our results also show how the independent firing of fate influencing TFs can robustly generate the regular proportions of all the different neuronal types within the retina from a pool of equipotent progenitors even though there is great clone-to-clone variability.

Despite its success, it is important to note that the model presented here has many limitations. First, it is a minimal model, meant only to cover the major classes of retinal neurons. There are, however, many neuronal subtypes of each major class that this model makes no attempt to account for. Second, to formulate this minimal model, we employed a reduced level of description, capturing only the core transcriptional network in the zebrafish retina. This reduction was essential in order to define a well-constrained and testable model that included biological mechanisms and just two free parameters that could be fitted to the means of a WT to WT dataset. It should therefore not be too surprising that such a minimal model does not match all the datasets perfectly. Interestingly, the few significant deviations between the predicted and the experimental distributions might be explainable by reasonable biological possibilities (see below). However, the capability of such a simple model to predict clonal statistics (clone composition, average clone sizes, and detailed size distributions) in so many contexts suggests that it might have essential validity, despite its limitations.

It is also important to note that this model does not address the fine scale structure of clonal distributions, especially the “tips” of lineages. Indeed, there are a number of cases, highlighted in a recent review, where terminal and penultimate divisions are biased toward particular outcomes (Cepko, 2014). Among these are the symmetric PR-PR, BC-BC, and HC-HC terminal divisions seen in the zebrafish retina (He et al., 2012). Some of these biases in late RPCs are clearly species specific, as BC-BC pairs are not common in mammals (Gomes et al., 2011). In the case of zebrafish, we suggest that it is the early RPCs in which the choice is apparently stochastic, i.e., at the beginning of neurogenesis, when RPCs sort themselves into one of four intermediary progenitor types (Atoh7+Ptf1a−, Atoh7−Ptf1a+, Atoh7+Ptf1a+, and Atoh7−Ptf1a−) via the independent probabilistic expression of these factors. Once this sorting period is over, each of these progenitor classes is endowed with a different potential.

In our previous work, eliminating particular cell types from the zebrafish retina, we have been struck by the small effect on the overall size of the retina (Almeida et al., 2014; Randlett et al., 2013) and that RPCs have a strong intrinsic potential to produce clones of a given mean size (He et al., 2012). In one extreme example, for instance, we used a combination of morpholinos, mutants, and pharmacological agents to generate a retina containing only two cell types, namely BCs and PRs (Randlett et al., 2013). Yet these retinas were only slightly smaller than WT retinas. The present model shows how clone size may be specified relatively independently of fate. In our model it is only the expression of Atoh7 that has an effect on clone size, as Atoh7, besides assigning GC fate, also influences the mode of RPC division (He et al., 2012). Thus, the model predicts changes in clone size distributions only when Atoh7 is knocked down, but these changes are relatively small, as Atoh7 is only expressed in a minority of RPCs during a brief temporal window. Interestingly, by transplanting WT cells into either Atoh7 or Ptf1a morphants, we also found an extrinsic effect on clone size, as WT RPCs developing in these environments tend to produce larger clones than they do in a WT environment. There is a potential biological explanation for this as both ACs and GCs are sources of Shh, a factor known to affect proliferation in the zebrafish retina (Locker et al., 2006; Shkumatava et al., 2004; Wall et al., 2009) and quicken the onset of neurogenesis (Shkumatava et al., 2004). It may therefore be that the reduced levels of Shh in both morphant environments cause a delay in the onset of neurogenesis, increasing clone sizes and biasing these lineages toward later fates.

Surprisingly, we did not observe the expected homeostatic compensations by WT RPCs in environments lacking GCs and ACs, as has been suggested in some other studies (Jusuf et al., 2011; Poggi et al., 2005; Yang, 2004). Instead, in our hands, WT RPCs in morphant environments did not overproduce cell types that were missing from that environment. What may account for these differences? In the previous studies, donor RPCs expressed only the cell-type specific transgenes, while in the present study, we use H2B-GFP to label all the cells in a clone and Ptf1a-DsRed to label all the ACs and HCs. Thus, for example, in the study of Poggi et al. (2005), it is possible that some of the Atoh7:GFP cells in the GC layer may have been misclassified as GCs when they were actually displaced ACs. In Atoh7 mutants and morphants, due to the absence of GCs, almost all the cells in the RGC layer are displaced amacrine cells (dACs). If these are misclassified as GCs, it would look like a significant increase in GCs. The current techniques would not allow such misclassifications. Confidence in the present findings comes from our ability here to count and identify all the cells, including displaced ACs, in large sets of individual clones, combined with the consistency of the statistical effects in morphants and mutants. The conclusion of minimal homeostatic compensation is independently supported by the fact that rat RPCs in clonal cultures give rise to clonal distributions that are similar to those in vivo (Cayouette et al., 2003); i.e., there is not an overproduction of GCs or ACs in these clones even though they are grown in the absence of any feedback cues.

The model outlined here has three phases. In the first of these phases, all cells are proliferative. The retina then enters two successive phases of neurogenesis. In the early neurogenic phase of mammalian embryos, mainly GCs, ACs/HCs and cone PRs are generated, while BCs and rod PRs largely appear in the late neurogenic phase. Previous studies, in a variety of vertebrates, have also suggested that there are two phases of retinal neurogenesis, with early cell types generated in the first and late cell types in the second (Elliott et al., 2008; Georgi and Reh, 2010; Morrow et al., 2008). Our model fits these findings well, but it posits that both the early and late neurogenic phases are stochastic in the sense that, within each phase, cells have fixed probabilities of expressing particular TFs and of leaving the cell cycle. An interesting question is what drives the cells through these phases. Recent work in mice has shown that Ikaros and Casz1, the vertebrate homologs of Hunchback and Castor, which control temporal identity in *Drosophila* CNS neuroblasts, may regulate the early and mid/late phases of retinal neurogenesis (Elliott et al., 2008; Mattar et al., 2015). It will be interesting to understand whether these factors drive the RPCs from one phase to the next and are themselves stochastically expressed as has recently been suggested (Barton and Fendrik, 2015).

The idea that TFs are probabilistically and independently expressed in retinal precursors means that there should be predictable populations of precursors that express certain combinations of TFs. This is similar to the idea that, in a large population of dice rolls, there will be a predictable number of snake eyes. Previous work has shown that specific cell fates in the retina may be greatly influenced by combinatorial coding mechanisms (Ohsawa and Kageyama, 2008; Wang and Harris, 2005). If a retinal precursor expressing particular combination of TFs is the product of the probability of expressing each TF individually, then the proportions of certain cell types should simply reflect this product of probabilities. Here, we show that this could explain why only about 3% of the cells in the retina are HCs, as these cells represent the intersection of independently expressed Ptf1a and Lhx1. Theoretically, this concept can explain how relatively few TFs could, from a pool of equipotent precursors, create a large and well-proportioned array of cell types and subtypes with high fidelity corresponding to the intersection sets of probabilistically expressed TFs.

There are other systems where stochastic phenomena regulate neural cell fates. For example, in the mouse olfactory epithelium, the choice of which receptor a sensory neuron expresses is partially stochastic. However, once one odorant receptor gene is expressed in a sensory cell, all other odorant receptor genes are repressed (Lomvardas et al., 2006). In the case of Dscam and clustered protocadherins, stochasticity is generated at the level of mRNA splicing rather than gene expression (Hattori et al., 2009; Lefebvre et al., 2012). In contrast to these systems, here we quantitatively account for the variability in clonal fates in the retina by a model in which the probabilities of expressing all, none, or any combination of these key fate determining genes is governed by the independent probability of expressing each of them individually. Recent studies into noisy gene expression systems show that stochastic mechanisms can indeed explain such probabilistic firing of genes in multicellular and microbial systems (Boettiger, 2013; Frank, 2013; Rister and Desplan, 2011). It has also been shown that fate determining bHLH TFs in mouse neural progenitor cells oscillate at rates much faster than the cell cycle (Imayoshi et al., 2013). Such oscillations in Atoh7 and Ptf1a, if asynchronous, might explain their independent probabilities of expression, but there are also several other reasonable possibilities such as gene position in the nucleus or epigenetic variability.

Whatever the molecular mechanisms may be in the case of the zebrafish retina, we show here that the high degree of variability in the lineages of RPC cells can be explained using a simple stochastic model based on these fixed probabilities of TF expression. It is important to note that, in this regard, whether or not a process is stochastic or follows some complicated deterministic rules is a matter of the level of description. Complex systems in which many variables interact often produce data that can best be described in terms of probabilities even though at the level of individual elements each of the interactions may be determinative. Statistically, however, stochastic processes produce robust and well-behaved distributions, as does the nervous system. This, we propose, is therefore a possible basis for understanding how it is that even though there is a high variability in the size and composition of individual clones, the total number of differentiated cells and the relative proportions of each cell type are almost invariant from one zebrafish retina to the next.

## Experimental Procedures

### Animals and Transgenic Lines

Zebrafish lines were maintained and bred at 26.5°C. Embryos were raised at 28.5°C or 32°C and staged as described previously in hpf (Kimmel et al., 1995). Embryos were treated with 0.003% phenylthiourea (PTU) (Sigma) from 10 hpf to prevent pigmentation. All procedures were performed under the project license PL80/2198 approved by the UK Home Office and by the Local Ethical Review Panel at the University of Cambridge. The transgenic lines used have all been described previously and are listed in Supplemental Information.

### Morpholino Injections

Antisense translation blocking morpholinos were obtained from Gene Tools, reconstituted as 1 or 3 mM stock solutions in water, and injected into the yolk at the one-cell stage. Morpholinos targeting Ptf1a, Atoh7, and Vsx1 have all been described previously and sequences are listed in Supplemental Information. Control embryos were injected with 2 ng of standard control morpholino from Gene Tools.

### Blastomere Transplantation

Embryos from H2B-GFP, Ptf1a-dsRed double transgenic zebrafish were dechorionated by pronase digestion (0.6 mg/ml; Sigma) and placed in agarose molds (Adaptive Science Tools), and one to five blastomeres were transplanted into an unlabeled embryo at 3.5 hpf using a flame-pulled glass capillary (Sutter instruments, #b100-50-10) connected to a 2 ml syringe. The host embryos were allowed to recover at 32°C overnight in agarose-coated dishes in order to catch up developmentally. At 24 hpf, embryos were anaesthetized by 0.04% MS-222 (Sigma) and screened on an upright fluorescent microscope where isolated GFP-positive RPCs could be identified. Position and number of cells were logged before the fish were placed in individual wells at 28.5°C. At 72 hpf, embryos were fixed for 1 hr in 4% PFA, the eye dissected out, and mounted in 1% low melting agarose (Sigma) for imaging.

### Confocal Image Acquisition and Analysis

Retinal clones or entire retinas were imaged under 60 × (NA = 1.30) or 30 × (NA = 1.05) silicon oil objectives on an inverted laser-scanning confocal microscope (Olympus FV1000) fitted with GaAsP detectors. Image analysis was performed using Volocity Software (Perkin Elmer). Based on nuclear position and absence or presence of the Ptf1a reporter gene, the different cell types could be scored. The identity of MCs was difficult to discern, and in many cases, these would be counted as BCs. However, when located among the ACs, MCs were easily spotted, but were still quantified as unknown.

### Cell Dissociation

Cell suspensions were prepared from freshly dissected retinal tissue from quadruple transgenic embryos (Atoh7-gapGFP; Ptf1a-dsRed; Lhx1-GFP; Crx-gapCFP). Quadruple positive embryos were, at 72 hpf, transferred to cold (4°C) Ca^2+^-free medium (116.6 mM NaCl, 0.67 mM KCl, 4.62 mM Tris; 0.4 mM EDTA [pH 7.8]) (Harris and Messersmith, 1992) supplemented with 100 μg/ml of heparin and 0.04% MS-222. Fifteen to 20 retinas were dissected and transferred to glass Petri dishes. Without disturbing the retinas, the Ca^2+^-free medium was removed, and 0.25% Trypsin-EDTA was added. After a 10-min incubation at 37°C, the trypsin was removed, and the retinas were mechanically dissociated by pipetting using flame-pulled glass Pasteur pipette. For confocal imaging, single-cell suspensions were plated into 35-mm imaging dishes, seeded for 1 hr at 28.5°C, followed by imaging.

### Modeling and Statistics

The essence of the model is explained in Results and detailed in Supplemental Experimental Procedures. Statistical methods, also detailed in Supplemental Experimental Procedures, are used to compare the experimental clonal distributions to distributions generated by computer according to the rules of the model (explained in the Results). Similarly the statistical analyses are straightforward except for the Goodness of Fit, which is described in Supplemental Experimental Procedures.

## Author Contributions

H.B. carried out all the experimental work with some help from S.D. S.R. carried out the bulk of the modeling and most of the statistical analyses. S.R., H.B., B.S., and W.A.H. worked to frame the essence of the model, and B.S. and W.A.H. supervised the study. H.B., W.A.H., S.R., and B.S. all contributed to writing the manuscript.

## Supplemental Information

Document S1. Supplemental Experimental Procedures and Figures S1–S7

Table S1. Entire Clone Set, Related to Figures 1 and 3 Ptf1a+ GC/PR/BC represents cells of these types that were labeled by the dsRed reporter gene. n, nasal; m, medial; t, temporal.

Table S2. Clone Summary, Related to Figure 3 Ptf1a+ GC/PR/BC represents cells of these types that were labeled by the dsRed reporter gene.

Table S3. Statistical Calculations, Related to Figures 1 and 3 p values indicating statistical significance between clonal datasets. The columns denote different cells types and the rows different morphant condition. A p value for a given row is the result of a comparison between either subsets of the given datasets (e.g., position in retina, one- and two-cell origin) or compared with WT to WT (clone sizes).

Table S4. Goodness of Fit, Related to Figures 2, 4, and 7 χ2 goodness-of-fit values comparing the predicted marginal distributions of each cell type with the empirical distributions. High values correspond to a good agreement, while values smaller than 0.05 indicate that the model is inconsistent with the experimental results for a specific cell type.

Table S5. Summary of Lhx1-GFP, Ptf1a-dsRed Clones, Related to Figure 6

Table S6. Photoconversion Clones, Related to Figure 7

Document S2. Article plus Supplemental information
