# Supplementary material for: The Independent Probabilistic Firing of Transcription Factors: A Paradigm for Clonal Variability in the Zebrafish Retina
Source: Dev Cell. 2015 Sep 14;34(5):532–43. doi: 10.1016/j.devcel.2015.08.011 (PMC4572358; doi:10.1016/j.devcel.2015.08.011)
Supplement: Document S1. Supplemental Experimental Procedures and Figures S1–S7 [file mmc1.pdf]

Developmental Cell

Supplemental Information

The Independent Probabilistic Firing of Transcription Factors: A Paradigm for Clonal Variability in the Zebrafish Retina

Henrik Boije, Steffen Rulands, Stefanie Dudczig, Benjamin D. Simons, and William A. Harris

Supplemental Figures

Figure S1, relates to Figure 2.

Analysis of wild type variables.

A) Fate composition in WT clones generated in nasal or temporal WT environment revealed no significant differences. B) Clone size of WT clones generated in nasal or temporal WT environment revealed no significant differences. C) Correlation of fate distribution and the injection of morpholinos revealed no systematic differences. D) Injection of morpholinos did not significantly affect clone size. Error bars depict SEM. GC, ganglion cell; AC, amacrine cell; dAC, displaced amacrine cell; HC, horizontal cell; PR, photoreceptor cell, BC, bipolar cell.

Figure S2, relates to Figure 4, 6 and 7.

Individual fate and clone size distributions.

A-N) The individual distributions of AC+HC, GC, PR, BC and clone size for the various treatments. A-E, H, L, N compare distributions to the intrinsic model, while F, G, I-K, M, N show models that incorporate the extrinsic effects. GC, ganglion cell; AC, amacrine cell; HC, horizontal cell; PR, photoreceptor cell, BC, bipolar cell.

Figure S3, relates to Figure 3 and 6.

The Ptf1a-lineage during various treatments.

A) Absolute number of HCs + ACs and Ptf1a positive cells within a clone. B) Percentage of HCs + ACs and Ptf1a positive cells within a clone. Error bars depict SEM. AC, amacrine cell; HC, horizontal cell.

Figure S4, relates to Figure 3.

Vsx2 BCs in Vsx1 morphants.

A) A WT Vsx2-GFP, Ptf1a-dsRed clone in a WT host. Arrow indicates a Vsx2 positive BC. Arrow head indicates a Vsx2 positive Muller glia cell. Hollow arrow heads indicate processes of the Muller glia cell. Inset show a magnified view of the stratification of the BC. B) A Vsx1 morphant Vsx2-GFP, Ptf1a-dsRed clone in a WT host. Inset show a magnified view of the stratification of the BCs. Scale bar in B equals 10 μm and is also valid for A. HC, horizontal cell; BC, bipolar cell; AC, amacrine cell.

Figure S5, relates to Figure 6.

Horizontal cell retrograde migration.

A) A WT Lhx1-GFP, Ptf1a-dsRed clone in a WT host. The inner plexiform layer (IPL) and HC layer are indicated for reference. B) A WT Lhx1-GFP, Ptf1a-dsRed clone in a host injected with a mixture of Ptf1a morpholinos. HCs fail the retrograde migration but the majority (>90%) of these HCs are located on the normal side of the IPL amongst the ACs. See Table S5. C) Depicts boxed region on B. D) A larger Lhx1-GFP, Ptf1a-dsRed clone in a Ptf1a morphant host where two out of four HCs failed their retrograde migration. E) A WT Lhx1-GFP, Ptf1a-dsRed clone in a Atoh7 morphant host illustrating HCs displaced in the ganglion cell layer. Here 100% of the ectopic HCs were located on the wrong side of the IPL. F) A mitotic HC displaced in the ganglion cell layer in an Atoh7 morphant suggests that migration to the HC layer is not required for the progression of the post-differentiative division than normally occurs once the migration is completed. G) Quantification of intrinsically WT Lhx1-GFP, Ptf1a-dsRed clones in WT or morphant hosts. There is a significant correlation (rho=0.53, p=0.00002) between a successful retrograde migration by the HCs and the number of ACs within the clone for the Ptf1a morphant environment. Roughly 20 clones were quantified for each defined size and treatment (See Supplementary table S5 for summary of clones). There was no difference in the migration of HCs in Vsx1 morphant hosts (Suppl. Table S5).

Figure S6, relates to Figure 6 and 7.

Extrinsic feedback in Lakritz fish and Ptf1a morpholino mixture and clones generated by photoconversion.

A) Comparison between WT clones in Atoh7 morphant and Lakritz mutants hosts, and WT clones in Ptf1a morphant and Ptf1a morpholino mixture hosts. B) Clone sizes for the clone set in A. C) Photoconversion of Kaede positive RPCs in WT and Atoh7 morphant embryos validates and corroborates the transplantation results with a loss of GCs alongside an increase in the number of PRs

and BCs. D) Clone sizes for the clone set in C reveals significantly larger clones in the Atoh7 morphants. E) The combined number of HCs and ACs in clones generated by photoconversion in Atoh7 morphants is significantly higher. F) The percentage of HCs and ACs in clones generated by photoconversion in Atoh7 morphants is not significantly different compared to control clones. G) Number of HCs and ACs, and Ptf1a positive cells, in WT clones in hosts injected with the Ptf1a morpholino mixture show that there is no compensatory increase in these cell types when clones develop in an environment lacking HCs and ACs. Error bars depict SEM. GC, ganglion cell; AC, amacrine cell; dAC, displaced amacrine cell; HC, horizontal cell; PR, photoreceptor cell, BC, bipolar cell.

Figure S7, relates to Figure 4.
Alternative models.
Two different alternative models that, were either positively or negatively interdependent, did a worse job at recapitulating the morphant data-sets.

Fig. S1

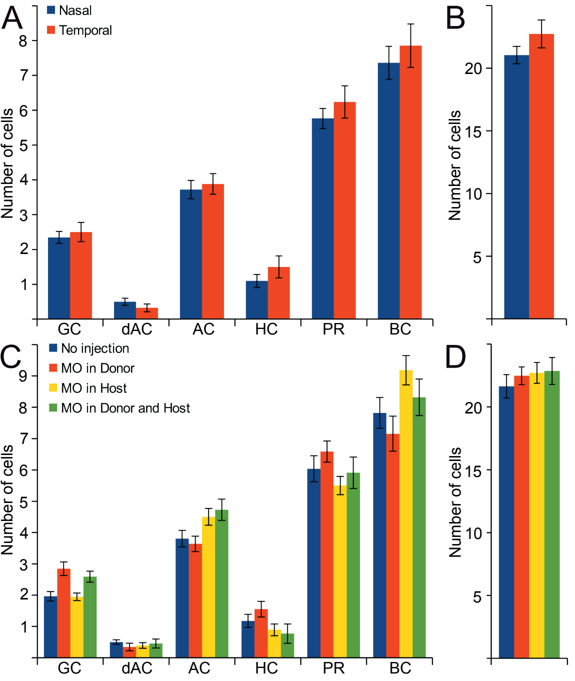
A
Nasal
Temporal
Number of cells
1
2
3
4
5
6
7
8
GC
dAC
AC
HC
PR
BC
B
Number of cells
5
10
15
20
C
No injection
MO in Donor
MO in Host
MO in Donor and Host
Number of cells
1
2
3
4
5
6
7
8
9
GC
dAC
AC
HC
PR
BC
D
Number of cells
5
10
15
20

Fig. S2

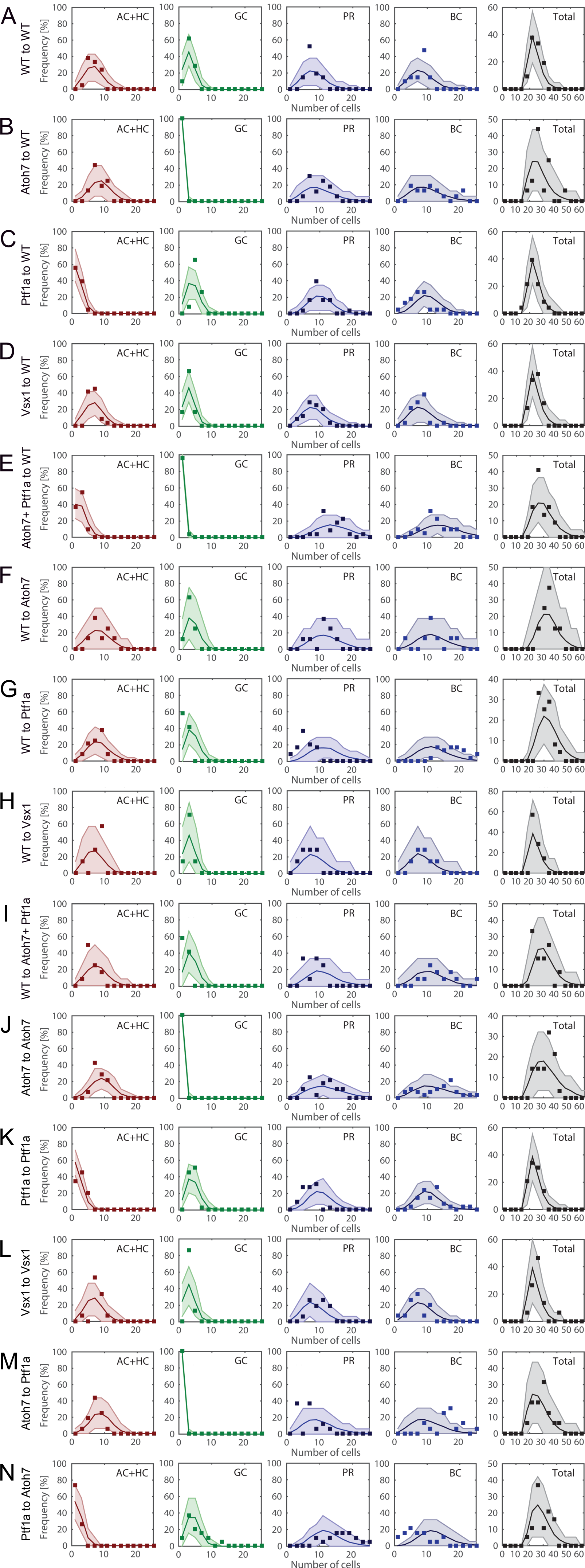

A
WT to WT
B
Atoh7 to WT
C
Ptf1a to WT
D
Vsx1 to WT
E
Atoh7+ Ptf1a to WT
F
WT to Atoh7
G
WT to Ptf1a
H
WT to Vsx1
I
WT to Atoh7+ Ptf1a
J
Atoh7 to Atoh7
K
Ptf1a to Ptf1a
L
Vsx1 to Vsx1
M
Atoh7 to Ptf1a
N
Ptf1a to Atoh7
AC+HC
GC
PR
BC
Total
Frequency [%]
Number of cells

Fig. S3

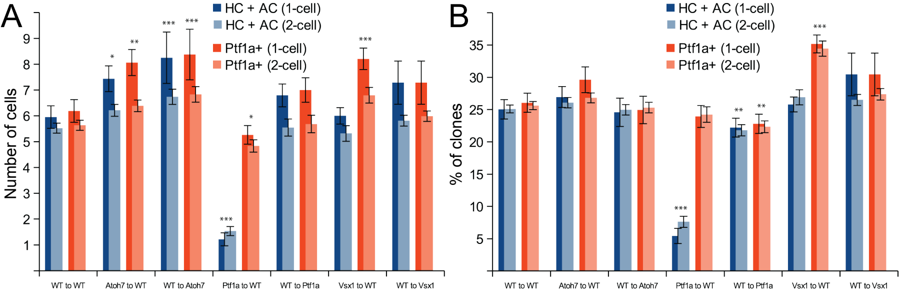
A
B
HC + AC (1-cell)
HC + AC (2-cell)
Ptf1a+ (1-cell)
Ptf1a+ (2-cell)
Number of cells
% of clones
WT to WT
Atoh7 to WT
WT to Atoh7
Ptf1a to WT
WT to Ptf1a
Vsx1 to WT
WT to Vsx1

Fig. S4

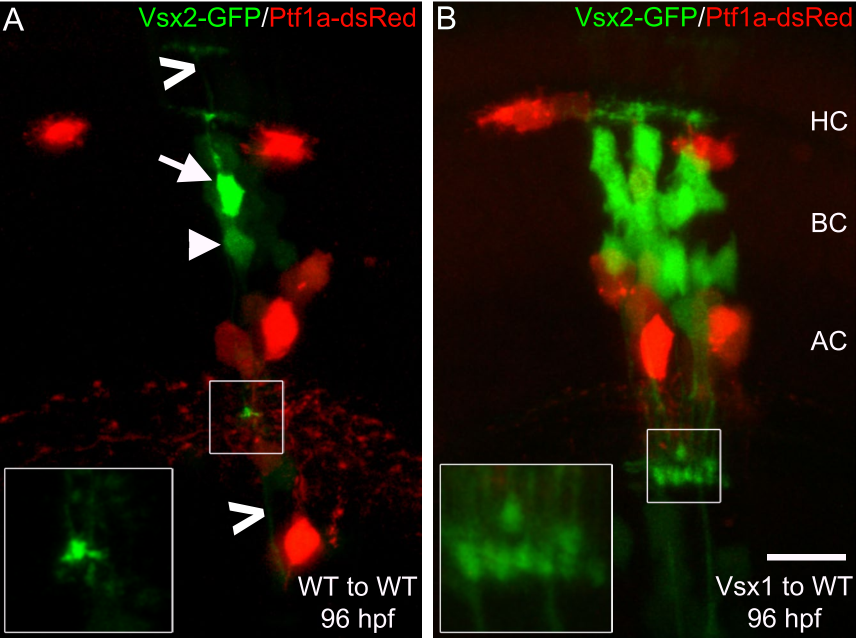

A
Vsx2-GFP/Ptf1a-dsRed
WT to WT
96 hpf
B
Vsx2-GFP/Ptf1a-dsRed
HC
BC
AC
Vsx1 to WT
96 hpf

Fig. S5

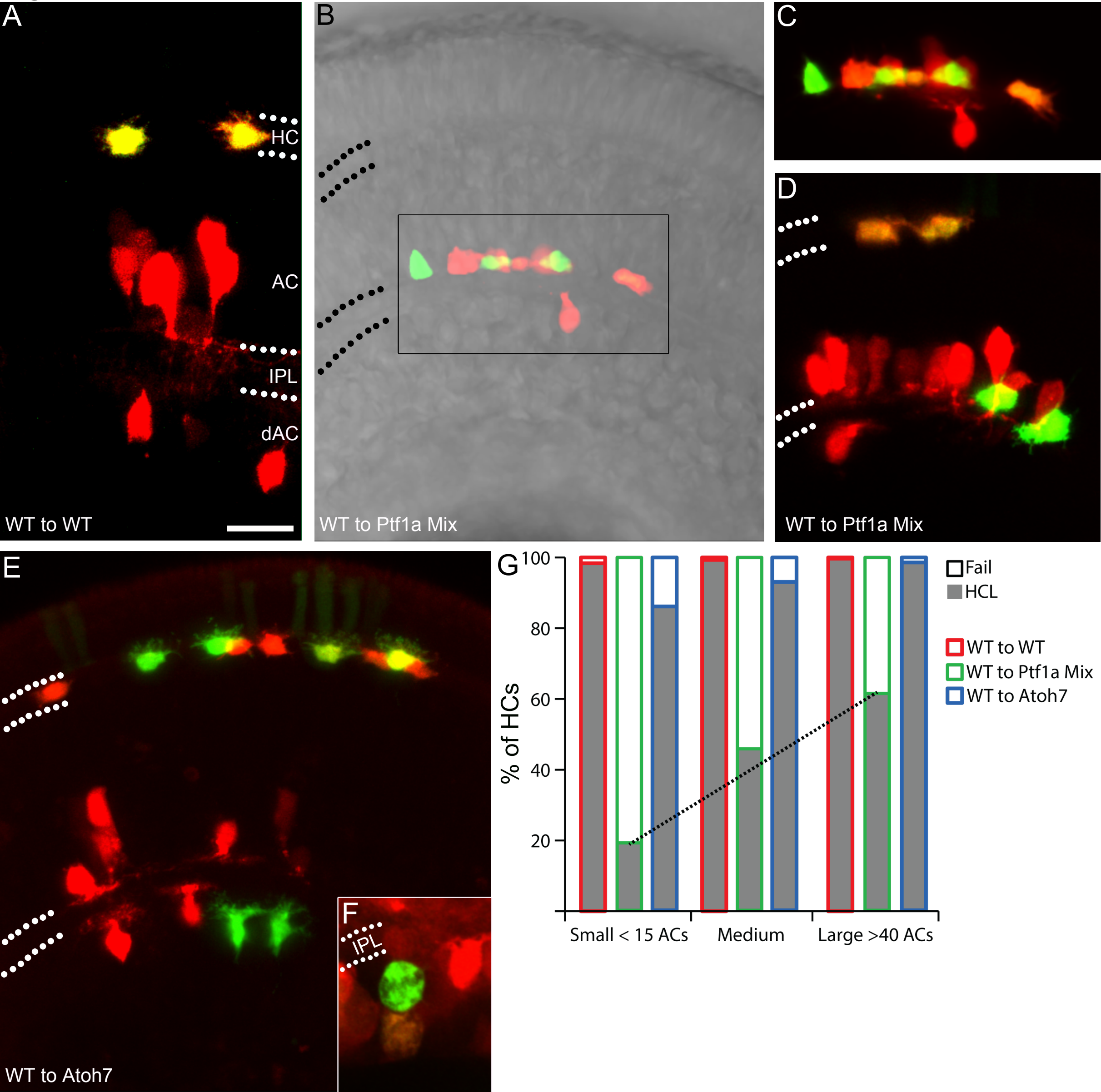
A
HC
AC
IPL
dAC
WT to WT
B
WT to Ptf1a Mix
C
D
WT to Ptf1a Mix
E
WT to Atoh7
F
IPL
G
100
80
60
40
20
% of HCs
Small < 15 ACs
Medium
Large >40 ACs
Fail
HCL
WT to WT
WT to Ptf1a Mix
WT to Atoh7

Fig. S6

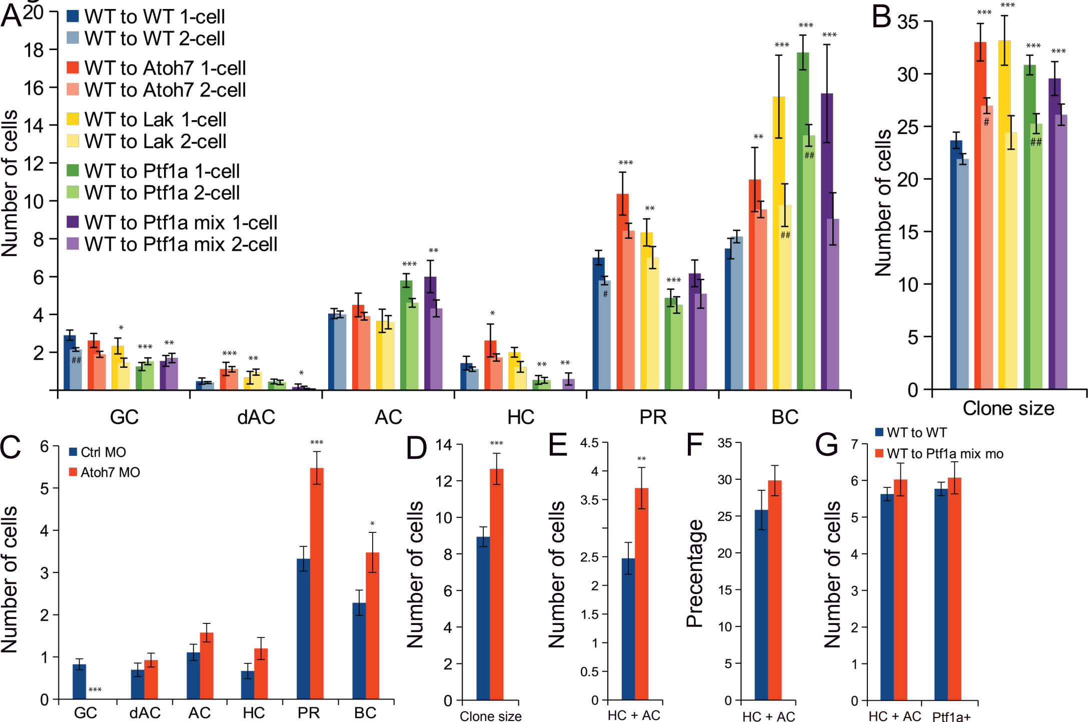

A
WT to WT 1-cell
WT to WT 2-cell
WT to Atoh7 1-cell
WT to Atoh7 2-cell
WT to Lak 1-cell
WT to Lak 2-cell
WT to Ptf1a 1-cell
WT to Ptf1a 2-cell
WT to Ptf1a mix 1-cell
WT to Ptf1a mix 2-cell
Number of cells
GC
dAC
AC
HC
PR
BC
B
Number of cells
Clone size
C
Ctrl MO
Atoh7 MO
Number of cells
GC
dAC
AC
HC
PR
BC
D
Number of cells
Clone size
E
Number of cells
HC + AC
F
Precentage
HC + AC
G
WT to WT
WT to Ptf1a mix mo
Number of cells
HC + AC
Ptf1a+

Fig. S7

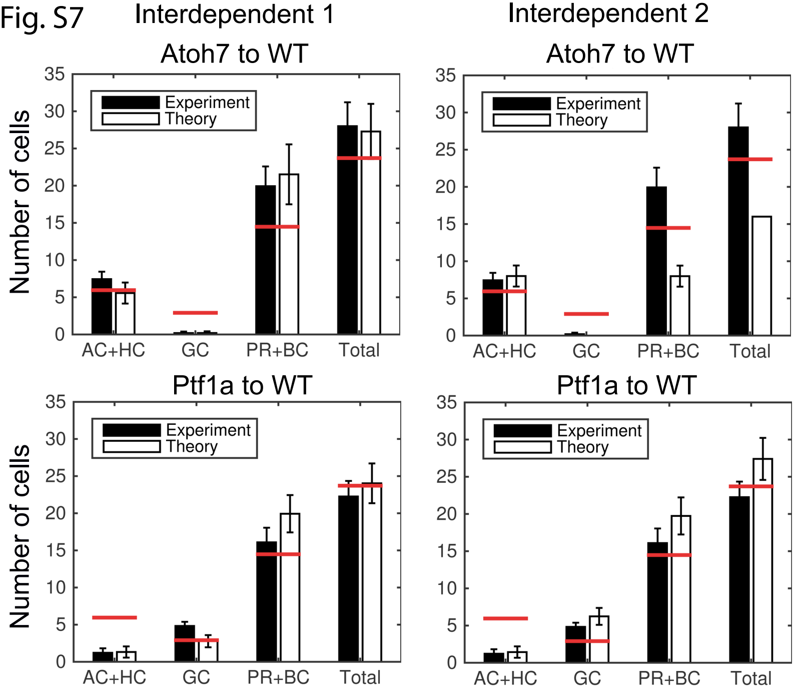

Interdependent 1

	AC+HC	GC	PR+BC
Ptf1a to WT	0.931062978	1.03E-09	0.001982874
Atoh7 to WT	4.74E-05	0.668921395	0.033720819

Interdependent 2

	AC+HC	GC	PR+BC
Ptf1a to WT	0.726425572	0.000634369	0.00376592
Atoh7 to WT	0.341688445	NaN	0.002456029

Description of Supplemental Tables

Table S1, relates to Figures 1 and 3: Entire clone set.

Abbreviations – GC, ganglion cells; dAC, displaced amacrine cells; AC, amacrine cells; HC, horizontal cell; PR, photoreceptor cell; BC, bipolar cell; n, nasal; m, medial; t, temporal. Ptf1a+ GC/PR/BC represents cells of these types that were labelled by the dsRed reporter gene.

Table S2, relates to Figure 3: Clone summary.

Abbreviations – GC, ganglion cells; dAC, displaced amacrine cells; AC, amacrine cells; HC, horizontal cell; PR, photoreceptor cell; BC, bipolar cell. Ptf1a+ GC/PR/BC represents cells of these types that were labelled by the dsRed reporter gene.

Table S3, relates to Figure 1 and 3: Statistical calculations.

p-values indicating statistical significance between clonal datasets. The columns denote different cells types and the rows different morphant condition. A p-value for a given row is the result of a comparison between either subsets of the given datasets (e.g. position in retina, 1 and 2 cell origin), or compared to WT to WT (clone sizes). Abbreviations – GC, ganglion cells; dAC, displaced amacrine cells; AC, amacrine cells; HC, horizontal cell; PR, photoreceptor cell; BC, bipolar cell.

Table S4, relates to Figures 2,4 and 7: Goodness of fit.

$\chi 2$ goodness of fit values comparing the predicted marginal distributions of each cell type with the empirical distributions. High values correspond to a good agreement, while values smaller than 0.05 indicate that the model is inconsistent with the experimental results for a specific cell type.

Table S5, relates to Figure 6: Summary of Lhx1-GFP, Ptf1a-dsRed clones.

Abbreviations – GC, ganglion cells; AC, amacrine cells; HC, horizontal cell; HCL, horizontal cell layer. Table S6: Photoconversion clones. Abbreviations – GC, ganglion cells; dAC, displaced amacrine cells; AC, amacrine cells; HC, horizontal cell; PR, photoreceptor cell; BC, bipolar cell.

Table S6 relates to Figure 7: Photoconversion clones.

Abbreviations – GC, ganglion cells; dAC, displaced amacrine cells; AC, amacrine cells; HC, horizontal cell; PR, photoreceptor cell; BC, bipolar cell.

Supplemental Experimental Procedures

Transgenic lines

Tg(H2B-GFP) and Tg(Ptf1a-dsRed) (He et al., 2012)

Tg(Lhx1-GFP) (Swanhart et al., 2010)

Tg(Vsx1-GFP) and Tg(Vsx2-GFP) (Kimura et al., 2008; Vitorino et al., 2009)

MAZe and UAS-Kaede transgenic lines (Collins et al., 2010; He et al., 2012)

Tg(Crx-gapCFP) (Suzuki et al., 2013)

Tg(Atoh7-gapGFP) (Jusuf et al., 2011)

Morpholino sequences

For Ptf1a, 12 ng morpholino targeted against the initiation codon (5-CCAACACAGTGTCCATTTTTTGTGC-3) was injected. For a more extensive knock down of Ptf1a, 12 ng of a second morpholino, targeted against a region 44 bp upstream from the translational start site (5-TTGCCCAGTAACAACAATCGCCTAC-3), was combined with the first Ptf1a morpholino and 2 ng p53 morpholino (5'- GCGCCATTGCTTTGCAAGAATTG-3'). These morpholinos have been described previously (Jusuf et al., 2011; Randlett et al., 2013). Co-injection of a p53-targeting morpholino limits off-targeting effects of morpholino injections at high concentrations (Bill et al., 2009). In order to prevent GC formation, 2ng of Atoh7 morpholino (5′TTCATGGCTCTTCAAAAAAGTCTCC-3′) was injected (Pittman et al., 2008). Knock-down of Vsx1 was accomplished by injection of 2 ng of Vsx1MO (5-AATTCACTTTCTCTCTCAGACTGGT-3) (Almeida et al., 2014). Control embryos were injected with 2 ng of standard control morpholino from Gene Tools.

Statistical analysis

We quantified clones of both one- and two-cell origin as we imagined that pairs of tightly juxtaposed labelled RPCs seen at 24 hpf had divided relatively recently from a single RPC. To test this idea, we compared clonal data generated from random pairs of one-cell origin clones to those of two-cell origin clones (Mann-Whitney U-test). The combined one-cell clones tended to be of larger than clones of two-cell origin, although this difference was not significant. There were, however, significantly fewer GCs and PRs in clones from two-cell origin (Fig. 3A, B). These results suggest that the two-cell origin clones are indeed likely to be recent daughters of a single RPC, as these differences described above likely reflect the fact that single RPCs in 24 hpf retinas are on average close to their next division while a pair of RPCs at 24 hpf have, on average, recently undergone a division. This temporal difference may affect competence windows and explain small differences in proliferation and fate determination. Statistical analyses on clones of one- and two-cell origin allowed us to calculate a p-value for each, which were then combined following Fisher's method (Fisher, 1925), and these values are reflected in the bar graphs (Figs. 3, 6, and 7). As this analysis shows that clones of one-cell origin and normalized clones of two-cell origin do significantly differ even though the two cell clones are likely to have a slightly earlier single cell origin, we decided to only use clones of one-cell origin for comparisons with the model predictions (see below). In all our statistical analyses, since the distributions of single cell types are non-normal we employed statistical tests not making any assumptions on the underlying probability distribution (Mann-Whitney U test when comparing two groups and Kruskal-Wallis test when comparing more than two groups). To compare the overall clone sizes we employed a t-test. During development, neurogenesis travels as a wave over the retina initiating nasal and finishing in the temporal parts. Our screening point prior to neurogenesis should circumvent this possible source of variation. Indeed, we found no significant difference in clone size or fate distribution between clones generated in different regions of the retina (Fig. S1A, B). To eliminate the injection of morpholinos as a cause of experimental variability, the WT clone set consisted of four groups where neither donor nor host were injected; either the donor or host were injected; or both donor and host were injected with the control morpholino. There were no significant trends between the different treatments (Fig. S1C, D).

Supplemental Theory

In order to understand the mechanisms of fate choice in the developing zebrafish retina we sought to define the simplest model that was consistent known the regulatory factors and the clonal data. We started by assuming independent probabilistic expression of certain nodal transcription factors and then refined this model by known regulatory mechanisms in order to describe fate choice under different morphant conditions. Specifically, we supposed that nodal TFs (Atoh7 and Ptf1a) are independently expressed such that, in each generation of the lineage, cells express Ptf1a and Atoh7 with specified probabilities, p_{Ptf1a} and p_{Atoh7}. According to their expression levels, cells fall into four groups: cells expressing Atoh7, but not Ptf1a, undergo an asymmetric (PD) division generating a GC. Cells expressing Ptf1a, but not Atoh7, undergo a terminal (DD) division resulting in two differentiated cells of type AC or HC. RPCs expressing both TFs divide asymmetrically producing either an AC or HC. Finally, RPCs lacking these TFs divide symmetrically, either undergoing a proliferative (PP) division or a DD division resulting in two late-born neurons, i.e. PR or BCs. The fraction of non-proliferative divisions creating late born neurons (given that they do not express Ptf1a or Atoh7) represents a third and final adjustable parameter in our model, p_{ng}. The TFs Vsx1 and Vsx2 mainly effect the PR and BC fates. Strikingly, the experimental data suggests an equal proportion of both cell types. We therefore suppose that the fate choice of differentiating cells, which do not express Ptf1a or Atoh7, is aptly described by a "coin flip" between the PR and BC fate. Relating division modes to TF expression in this way yields the probabilities of a single cell undergoing PP, PD, or DD divisions as a function of the model parameters p_{Ptf1a}, p_{Atoh7}, and p_{ng} ,

$$p_{PP} = (1 - p_{Atoh7})(1 - p_{Ptf1a})(1 - p_{ng}),$$

$$p_{PD} = p_{Atoh7},$$

$$p_{DD} = 1 - p_{PP} - p_{PD}.$$

Previous studies suggest that the mode of cell division varies temporally (He et al., 2012), which indicates that the expression levels of Ptf1a and Atoh7 are also time-dependent. Motivated by these investigations, we therefore considered three temporally distinct regimes of TF expression: In the first three generations of a lineage, none of the differentiation factors are expressed. Then, for two rounds of division, all of these factors can be expressed, while after that only the parameter p_{ng} remains non-zero, cf. Fig. 2B. Taking the time dependence of TF expression into account, the probabilities of division modes, p_{PP}, p_{PD}, and p_{DD} follow closely the time dependencies proposed in recent studies (He et al., 2012).

Mathematically, the time-evolution of cell numbers is then described by a Galton-Watson birth-death type process with time-dependent rates. For a given mode of differentiation (PD or DD) and given numbers of PD and DD divisions in a lineage, N_{DD} and N_{PD}, cell numbers are specified by the probabilities to become a given cell type during a single division. For example, the probability for a Ptf1a expressing cell (AC or HC) to be derived through a DD type division is given by

$$p_{Ptf1a|DD} = \frac{p_{Ptf1a}(1 - p_{Atoh7})}{p_{DD}}.$$

Similarly, the probability of a PR to arise from a DD division is

$$p_{PR|DD} = (1 - p_{Ptf1a})(1 - p_{Atoh7})p_{ng}/(2p_{DD}),$$

and consequently

$$p_{BC|DD} = 1 - p_{Ptf1a|DD} - p_{PR|DD.}$$

Accordingly, for cells arising from PD divisions we find

$$p_{Ptf1a|PD} = \frac{p_{Ptf1a}p_{Atoh7}}{p_{PD}},$$

$$p_{GC|PD} = 1 - p_{Ptf1a|PD.}$$

Let us now define the numbers $k_{GC}, k_{AC+HC}, k_{BC}$, and k_{PR} of cells of each type that arise from a given number of DD divisions, N_{DD}. Then, noting that cells arising from DD divisions are created pairwise, the numbers $k_{GC}, k_{AC+HC}, k_{BC}$, and k_{PR} are determined by the distribution of the numbers of the parents of these terminal divisions, $k_{GC}/2, k_{AC+HC}/2, k_{BC}/2$, and $k_{PR}/2$. If these differentiation events are statistically independent this distribution is multinomial, i.e.

$$P_{DD}(k_{GC}, k_{AC+HC}, k_{BC}, k_{PR}|N_{DD}) = \frac{N_{DD}!}{(k_{GC}/2)!\,(k_{AC+HC}/2)!\,(k_{BC}/2)!\,(k_{PR}/2)!} p_{GC|DD}^{k_{GC}/2} p_{Ptf1a|DD}^{k_{Ptf1a}/2} p_{PR|DD}^{k_{PR}/2} p_{BC|DD}^{k_{BC}/2},$$

if all $k > 0$ (i.e. for each cell type), or 0 otherwise. Of course, the total number of cells arising from DD division, $k_{GC} + k_{AC+HC} + k_{BC} + k_{PR}$ must not exceed 2 N_{DD}. Similarly, the cell number arising from PD divisions, N_{PD}, are distributed according to

$$P_{PD}(k_{GC}, k_{AC+HC}|N_{PD}) = \frac{N_{PD}!}{k_{GC}!\,k_{AC+HC}!} p_{GC|PD}^{k_{GC}} p_{Ptf1a|PD}^{k_{Ptf1a}}.$$

With these definitions, marginalization over the distributions of the number of DD or PD divisions yields the overall distributions that arise from these divisions:

$$P_{DD}(k_{GC}, k_{AC+HC}, k_{BP}, k_{PR}) = \sum_{N_{DD}} P_{DD}(k_{GC}, k_{AC+HC}, k_{BC}, k_{PR}|N_{DD})P(N_{DD}),$$

$$P_{PD}(k_{GC}, k_{AC+HC}) = \sum_{N_{PD}} P_{PD}(k_{GC}, k_{AC+HC}|N_{PD})P(N_{PD}).$$

Finally, the overall distribution of the numbers of cell types can then be obtained by computing the convolution of P_{DD} and P_{PD}. We calculated $P(N_{DD})$ and $P(N_{PD})$ by Monte Carlo simulations of the time dependent Galton-Watson process. The convolution was computed by random sampling from the resulting distributions.

Building upon this minimal model for WT clones in WT environments, we then extended this model in order to accurately describe the changes that occur upon knock down of TFs. To this end, we took into account known details of the transcriptional network. Since, in the absence of Atoh7, asymmetric divisions generating a GC are replaced with symmetric proliferative divisions (He et al., 2012; Poggi et al., 2005), the probability of undergoing PP divisions is effectively increased by the probability of not expressing Ptf1a times the efficiency of knocking down Atoh7: $\Delta_{\mathrm{Atoh7}}(1 - p_{Ptf1a})p_{ng}$ with the reduction in Atoh7 expression probability defined by $\Delta_{Atoh7} = p_{Atoh7}^{WT} - p_{Atoh7}$. On the other hand, in

the case of Ptf1a knockdown, the total clone size remains unchanged such that, in this case, the proliferative aspects remain unchanged. Specifically, we ensured that the probabilities of PP, PD and DD divisions remain unchanged upon changes of p_{Ptf1a} by adding the term $\Delta_{Ptf1a}(1-p_{Atoh7})(1-p_{ng})$ to the probability of PP divisions.

To describe the effects of Vsx1 and Vsx2 in the case of TF knockdown, it turned out to be sufficient to ensure that, once a RPC not expressing Atoh7 or Ptf1a is licensed to differentiate, it has a 50% chance to become a PR. To this end, the ratio of DD divisions leading to a pair of PRs was increased by $\left[\Delta_{Ptf1a}\,(1-p_{Atoh7})(1-p_{ng})-\Delta_{Atoh7}\,(1-p_{Ptf1a})p_{ng}\right]/2$. Taken together, the probabilities of undergoing PP, PD, or DD divisions take the form

$$\begin{aligned}
p_{PP} &= (1-p_{Atoh7})(1-p_{Ptf1a})(1-p_{ng}) \\
&\quad +\Delta_{\mathrm{Atoh7}}(1-p_{Ptf1a})p_{ng}+\Delta_{Ptf1a}(1-p_{Atoh7})(1-p_{ng}), \\
p_{PD} &= p_{Atoh7}, \\
p_{DD} &= 1-p_{PP}-p_{PD}.
\end{aligned}$$

For the ratios of DD divisions that lead to specified cell types we find

$$\begin{aligned}
p_{Ptf1a|DD} &= \frac{p_{\mathrm{Ptf1a}}(1-p_{Atoh7})}{p_{DD}}, \\
p_{PR|DD} &= \left[(1-p_{Ptf1a})(1-p_{Atoh7})p_{ng}-\Delta_{Atoh7}\,(1-p_{Ptf1a})\,p_{ng}\right. \\
&\qquad \left.+\Delta_{Ptf1a}(1-p_{Atoh7})(1-p_{ng})\right]/(2\mathrm{p_{DD}}), \\
p_{BC|DD} &= 1-p_{Ptf1a|DD}-p_{PR|DD.}
\end{aligned}$$

Similarly, for PD divisions, we obtain

$$\begin{aligned}
p_{Ptf1a|PD} &= \frac{p_{Ptf1a}p_{Atoh7}}{p_{PD}}, \\
p_{GC|PD} &= 1-p_{Ptf1a|PD.}
\end{aligned}$$

With this modelling framework, we then estimated the parameters p_{Ptf1a}, p_{Atoh7}, and p_{ng}. To begin, we made use of existing live-imaging data and the previously published model predicting clone sizes (He et al., 2012). Based on the results of this earlier study, we took the probability of DD divisions as $p_{ng}=0.8$. We were left with two parameters to be determined by statistical inference. To make sure that we base our analysis on a statistically uniform population of clones, we focussed our analysis on clones of single cell origin alone. In order to reduce the influence of outliers we estimated the remaining two parameters by minimizing the sum of the squared errors between the mean cell numbers predicted by the model and the corresponding mean values in the experimental data employing a Nelder-Mead algorithm. Confidence regions were defined as the set of parameters for which all theoretical means do not differ significantly from the experimental means according to a t-test.

To describe morphants transplanted into wild-type retinas we assumed that the reduction in the expression probability in a given morphant is well estimated by the reduction in the numbers of cells of the corresponding cell type. For example, in the Atoh7 to WT experiment, the average number of GCs

is reduced by roughly 93%. Reducing the probability of expressing Atoh7 by the same amount, we found that $p_{Atoh7} = 0.02$. Similarly, the probability of expressing Ptf1a in the Ptf1a morphant to WT experiment was reduced by 79%, which translates to $p_{Ptf1a} = 0.06$. To further test our model we then predicted intrinsic effects in the double morphant. To this end we reduced both TF expression probabilities independently by the same amounts calculated for the single morphants.

We were able to describe many of the changes that occur in extrinsic treatments by merely postponing the onset of neurogenesis, while keeping the maximum expression levels and the total expression over the time course unchanged. Specifically, the assumption that 60% of cells postpone neurogenesis by one round of division in single morphant environments (40% of cells in the double morphant) favourably predicts the distributions of clone sizes and fate outcomes. To model clone formation in morphant-to-morphant transplantations, we simply reduced expression probabilities as in the intrinsic treatments, and took into account the effects of the extrinsic environment by changing the time point of the onset of neurogenesis in Atoh7 hosts, where 20% of cells postpone neurogenesis by one round of division.

To assess the degree to which our model predictions reproduce the experimental data, we calculated the probability that experimental deviations from the theoretical distributions (or any more extreme deviation) were the result of pure chance. A high probability indicates a good fit, while a probability below 0.05 would generally be an indication of a non-fitting model. Specifically, we employed a chi-squared goodness of fit test comparing observed and predicted marginal distributions of each cell type. The squared errors between both distributions are known to follow a chi-squared distribution, which allowed us to calculate the p-value for our fits using Matlab's ® Statistics Toolbox™. The p-values are summarized in Table S4.

To test whether there are different model paradigms that can describe the experimental data we compared our model to two alternative models in which there is interdependent expression of the TFs. The ratio of BCs and PRs, which is linked to the expression of Vsx1/2, remains constant at ½ throughout all intrinsic experiments. This strongly suggests that Vsx1/2 expression is independent of Ptf1a and Atoh7. Since interdependence of Vsx1/2 with Ptf1a or Atoh7 would therefore trivially lead to advantage of our model, we combined PRs and BCs in this analysis.

Model 1: In the first model we assumed that expression of Ptf1a and Atoh7 are positively interdependent, i.e. knockdown of Ptf1a leads to a knockdown in Atoh7, and vice versa. On the functional level this means that cells that would have expressed a knocked-down TF cannot express the other TF.

Model 2: In the second model we assumed that expression of Ptf1a and Atoh7 are negatively interdependent, i.e. knockdown of Ptf1a leads to the overexpression of Atoh7, and vice versa. On the functional level this means that cells that would have expressed a knocked-down TF express the other TF instead.

As shown in Fig. S7 these alternative models cannot account for the changes that occur during single or double knockdown of TFs. This suggests that with our model we have identified simplest possible description of fate regulation in retinal progenitors, which is compatible with the experimental data.

Heat shock and Photoconversion

The MAZe line was crossed with the UAS-Kaede line. Embryos were collected and kept at 28°C. At 8 hpf, a brief heat shock was applied at 39°C for 1 min. After 12 hr, the heat-shocked embryos were screened on an upright fluorescent microscope and the retinas with Kaede-expressing cells were selected. At 32 hpf, embryos were embedded in 3% methylcellulose (Sigma) and the green clones were found using a 60x water objective (NA = 1.3) on a spinning-disc microscope (Perkin Elmer). Single cells from the green clones were then randomly targeted and photoconverted by applying a 5 s train of 405 nm laser pulses. At 72 hpf these embryos were fixed and imaged as previously described.

Supplemental References

Almeida, A.D., Boije, H., Chow, R.W., He, J., Tham, J., Suzuki, S.C., and Harris, W.A. (2014). Spectrum of Fates: a new approach to the study of the developing zebrafish retina. Development (Cambridge, England) *141*, 1971-1980.

Bill, B.R., Petzold, A.M., Clark, K.J., Schimmenti, L.A., and Ekker, S.C. (2009). A primer for morpholino use in zebrafish. Zebrafish *6*, 69-77.

Collins, R.T., Linker, C., and Lewis, J. (2010). MAZe: a tool for mosaic analysis of gene function in zebrafish. Nature methods *7*, 219-223.

He, J., Zhang, G., Almeida, A.D., Cayouette, M., Simons, B.D., and Harris, W.A. (2012). How variable clones build an invariant retina. Neuron *75*, 786-798.

Jusuf, P.R., Almeida, A.D., Randlett, O., Joubin, K., Poggi, L., and Harris, W.A. (2011). Origin and determination of inhibitory cell lineages in the vertebrate retina. The Journal of neuroscience : the official journal of the Society for Neuroscience *31*, 2549-2562.

Kimura, Y., Satou, C., and Higashijima, S. (2008). V2a and V2b neurons are generated by the final divisions of pair-producing progenitors in the zebrafish spinal cord. Development (Cambridge, England) *135*, 3001-3005.

Pittman, A.J., Law, M.Y., and Chien, C.B. (2008). Pathfinding in a large vertebrate axon tract: isotypic interactions guide retinotectal axons at multiple choice points. Development (Cambridge, England) *135*, 2865-2871.

Randlett, O., MacDonald, R.B., Yoshimatsu, T., Almeida, A.D., Suzuki, S.C., Wong, R.O., and Harris, W.A. (2013). Cellular requirements for building a retinal neuropil. Cell reports *3*, 282-290.

Suzuki, S.C., Bleckert, A., Williams, P.R., Takechi, M., Kawamura, S., and Wong, R.O. (2013). Cone photoreceptor types in zebrafish are generated by symmetric terminal divisions of dedicated precursors. Proceedings of the National Academy of Sciences of the United States of America *110*, 15109-15114.

Swanhart, L.M., Takahashi, N., Jackson, R.L., Gibson, G.A., Watkins, S.C., Dawid, I.B., and Hukriede, N.A. (2010). Characterization of an lhx1a transgenic reporter in zebrafish. The International journal of developmental biology *54*, 731-736.

Vitorino, M., Jusuf, P.R., Maurus, D., Kimura, Y., Higashijima, S., and Harris, W.A. (2009). Vsx2 in the zebrafish retina: restricted lineages through derepression. Neural development *4*, 14.
